# Supplementary material for: Synthesis and Antibacterial Evaluation of Novel 1,3,4-Oxadiazole Derivatives Containing Sulfonate/Carboxylate Moiety
Source: Molecules. 2020 Mar 25;25(7):1488. doi: 10.3390/molecules25071488 (PMC7180883; doi:10.3390/molecules25071488)
Supplement: Supplementary file 1 [file molecules-25-01488-s001.pdf]

Synthesis and Antibacterial Evaluation of Novel 1,3,4-Oxadiazole Derivatives Containing Sulfonate/Carboxylate Moiety

Lei Wang, Xia Zhou*, Hui Lu, Xianfu Mu, Linhong Jin*

State Key Laboratory Breeding Base of Green Pesticide and Agricultural Bioengineering, Key Laboratory of Green Pesticide and Agricultural Bioengineering, Ministry of Education, Guizhou University, Huaxi District, Guiyang 550025, China. wanglei880328@163.com(L.W.); luhui2624904231@163.com (H.L.); Mu_xianfu@163.com (X.M.);

*Correspondence authors: E-mail: linhong_j@126.com, zhouxia_j@126.com; Tel.: (+86(851)3620521, Fax.: +868513622211).

Supplemental Materials

1. *In Vitro* Antibacterial Activity

Table1. EC_{50} (μM) of some target compounds against *Xanthomonas oryzae pv. oryzae* and *Xanthomonas axonopodis pv. citri* [a]

Compd.	*Xanthomonas oryzae pv. oryzae*			*Xanthomonas axonopodis pv. citri*		
	EC_{50} (μM)	regression equation	r	EC_{50} (μM)	regression equation	r
**4a-1**	63.4±3.8	y = 2.70x + 0.13	0.988	114.0±6.6	y=1.85x + 1.18	0.977
**4a-2**	50.1±4.2	y = 3.21x - 0.45	0.988	95.8±4.6	y=2.10x + 1.04	0.984
**4a-3**	87.2±4.7	y = 2.87x - 0.58	0.964	132.5±7.5	y=1.56x + 1.69	0.976
**4a-4**	99.4±4.7	y = 3.11x - 1.22	0.966	155.2±5.8	y=1.32x + 2.10	0.979
**4a-11**	98.0±6.6	y = 3.22x - 1.41	0.953	/	/	/
**4a-12**	95.3±3.9	y = 3.15x - 1.23	0.954	/	/	/
**4a-13**	86.4±4.8	y = 3.22x - 1.23	0.951	/	/	/
**4a-14**	69.0±4.4	y = 3.52x - 1.47	0.976	/	/	/
**4a-15**	83.4±6.0	y = 3.32x - 1.37	0.990	/	/	/
**4a-16**	112.5±6.0	y = 2.60x - 0.33	0.946	/	/	/
Bismerthiazol [b]	253.5±7.6	y = 2.03x + 0.12	0.969	274.3±8.6	y=1.76x + 0.70	0.978
Thiodiazole copper [b]	467.4±15.5	y = 1.78x + 0.23	0.979	406.3±13.0	y=0.92x + 2.60	0.983

[a] The statistical analysis was conducted by ANOVA method at the condition of equal variances assumed ($p > 0.05$) and equal variances not assumed ($p < 0.05$); [b] The commercial agricultural antibacterial agents Bismerthiazol, and Thiodiazole copper were used as positive control.

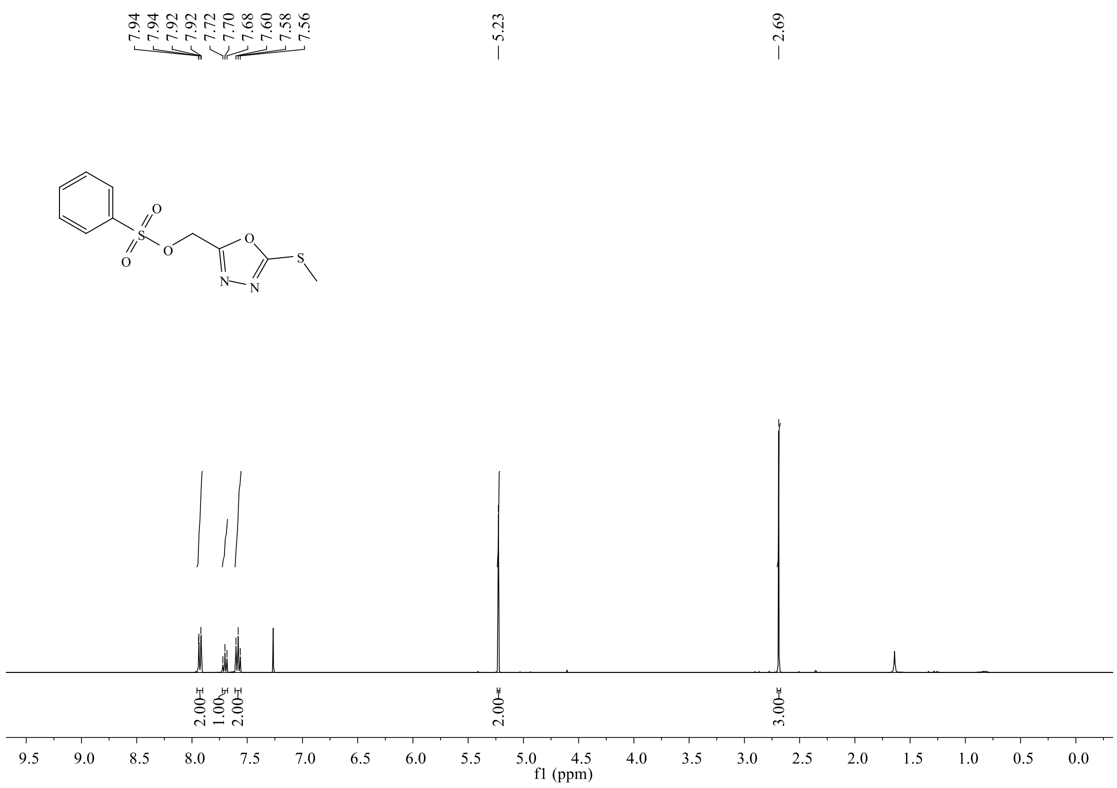

^{1}H NMR of compound **4a-1**

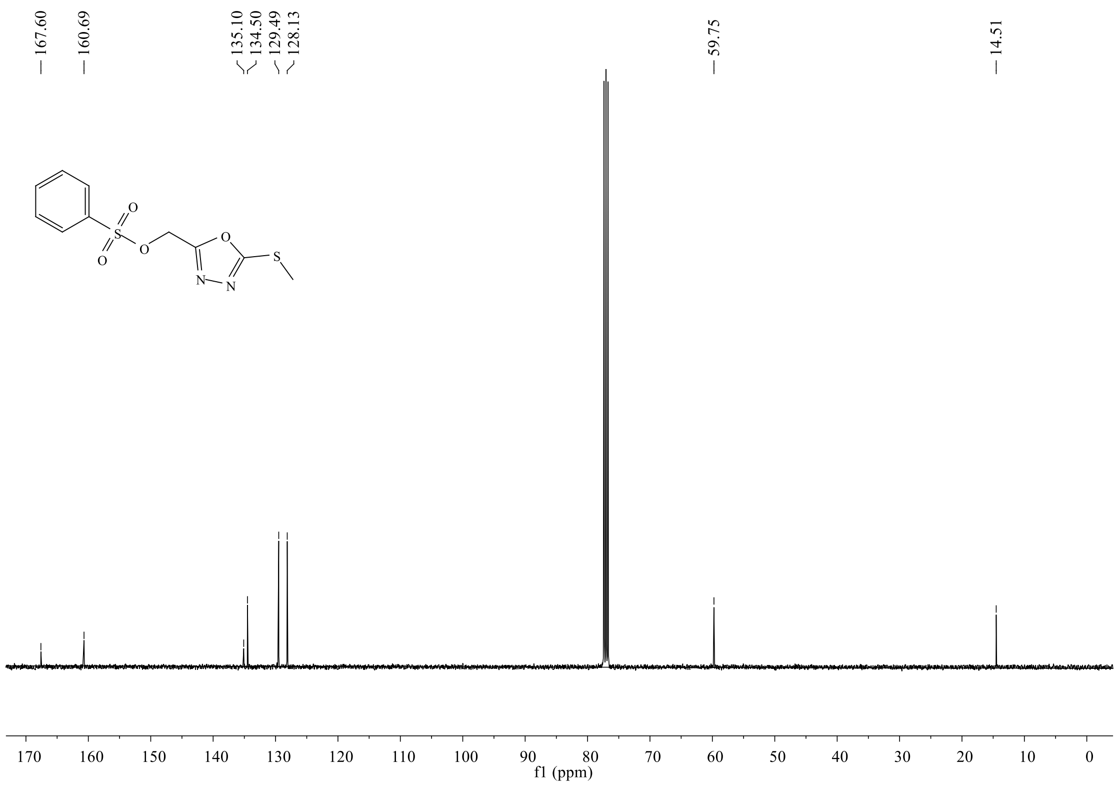

^{13}C NMR of compound **4a-1**

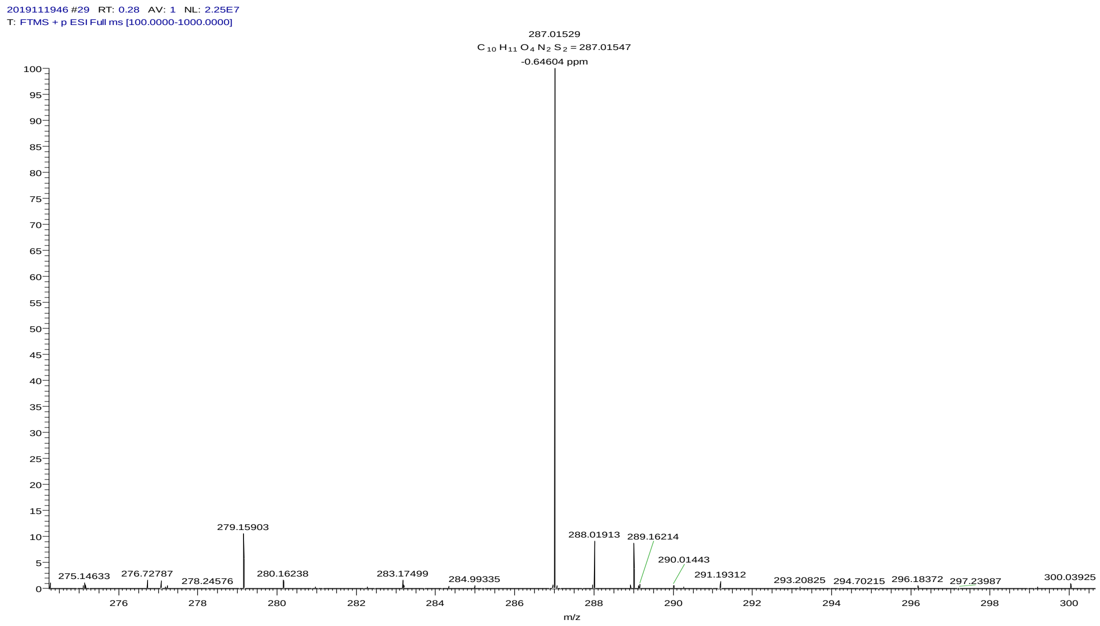

HRMS of compound **4a-1**

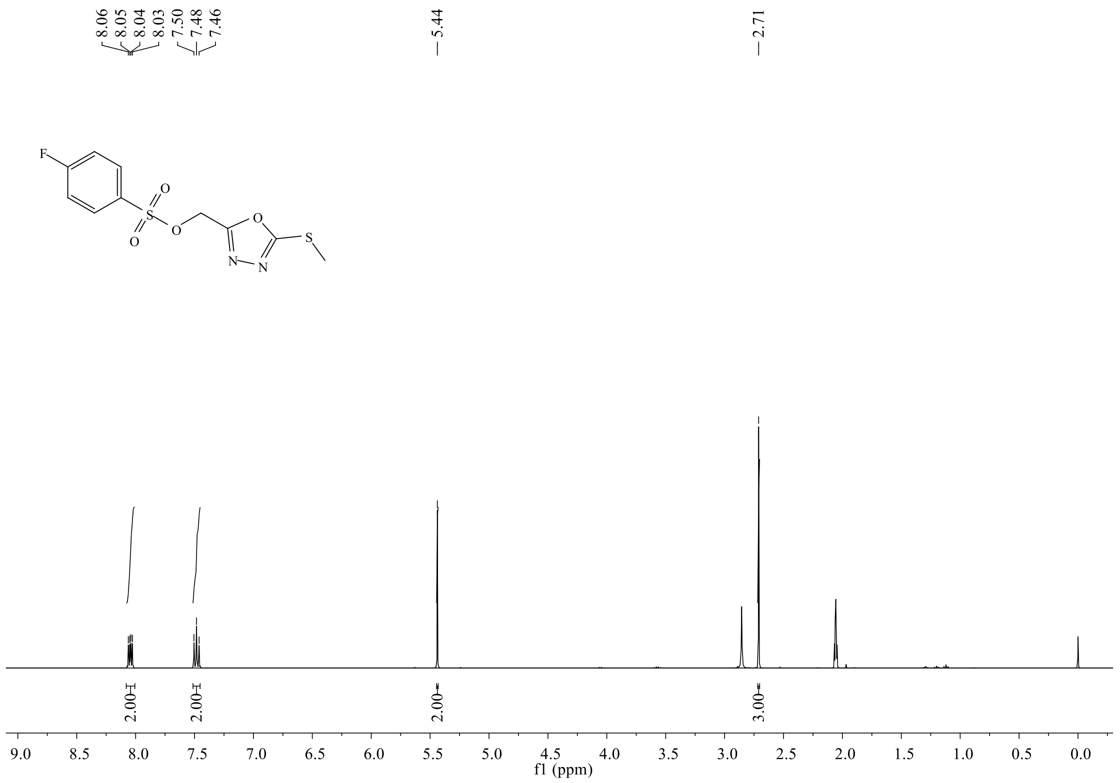

^{1}H NMR of compound **4a-2**

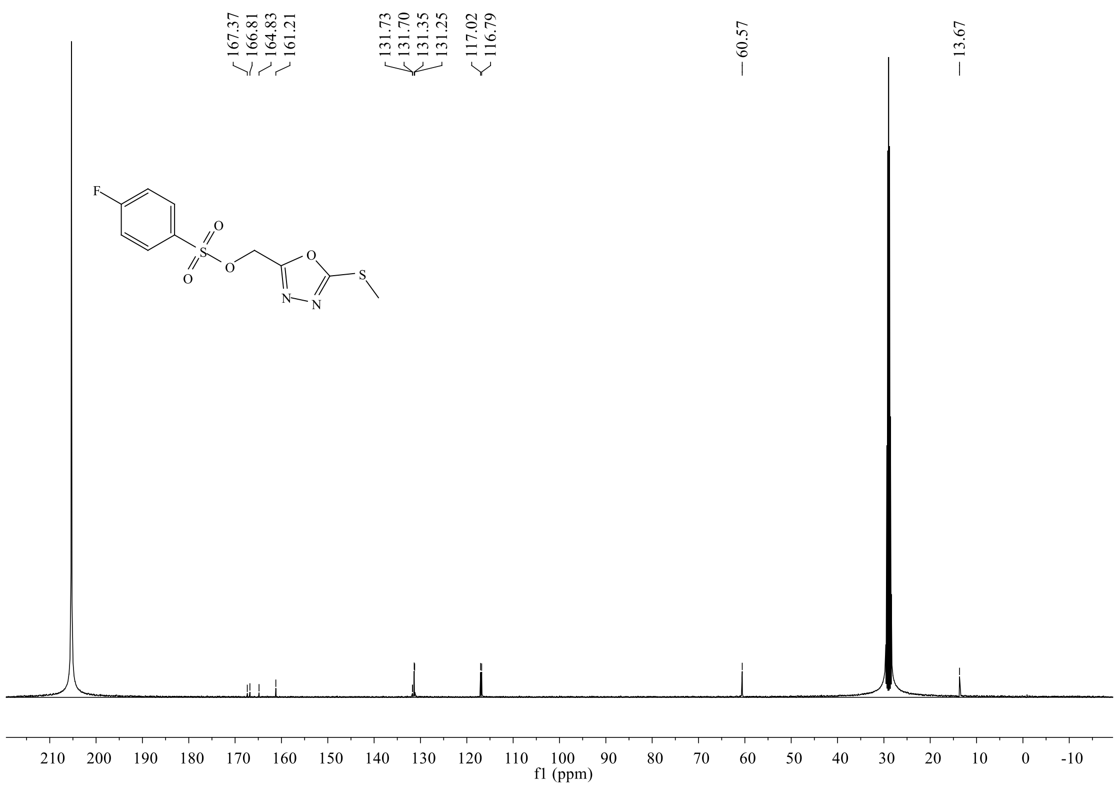

^{13}C NMR of compound **4a-2**

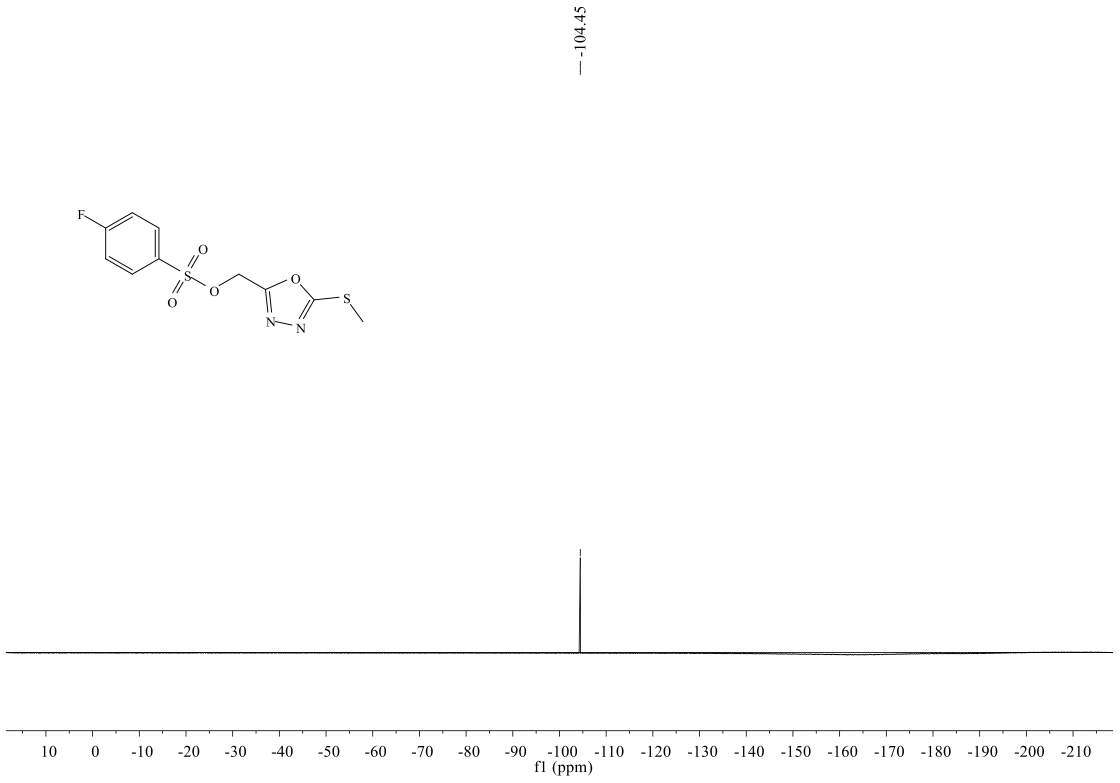

^{19}F NMR of compound **4a-2**

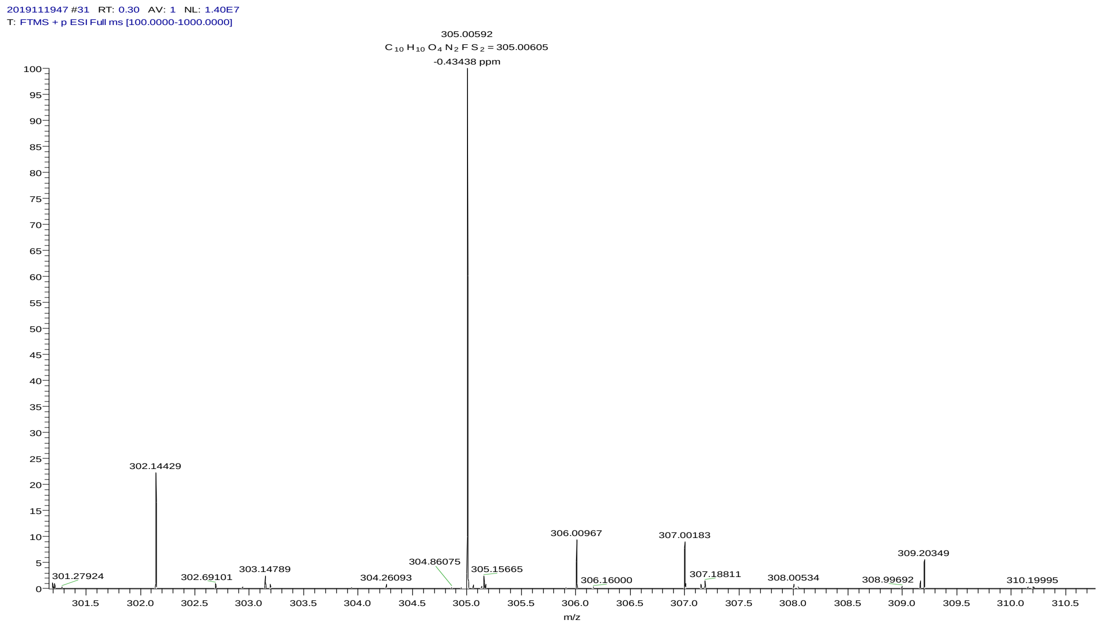

HRMS of compound **4a-2**

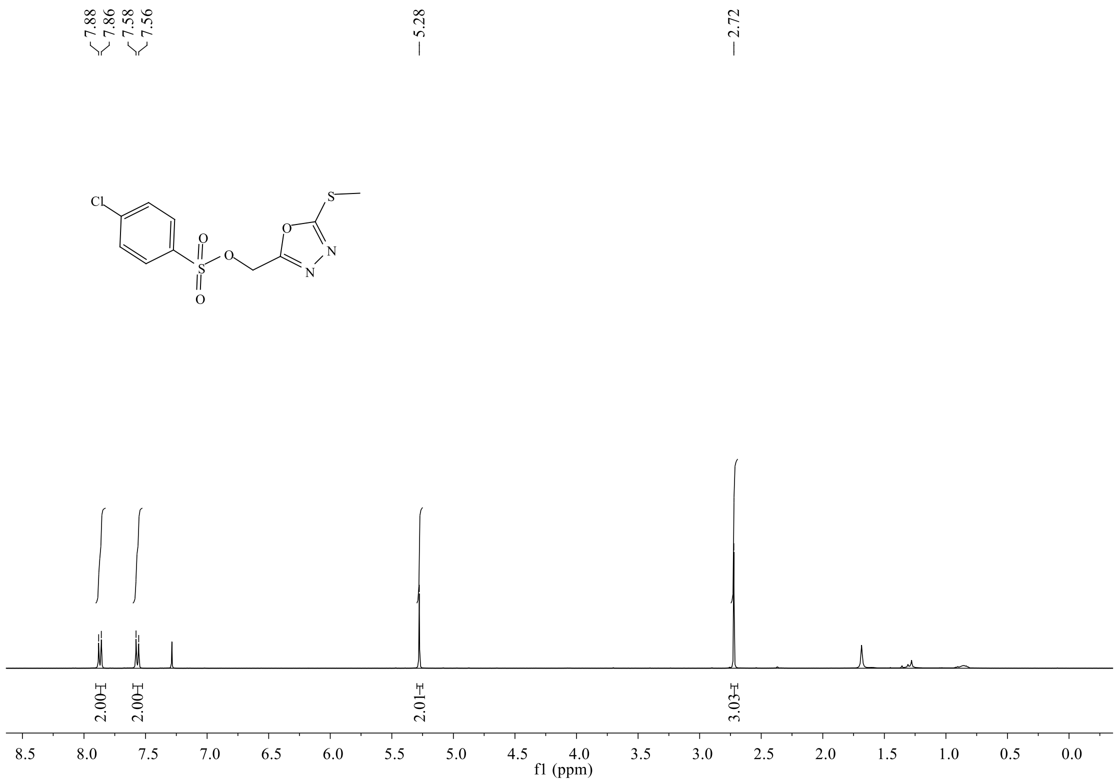

^{1}H NMR of compound **4a-3**

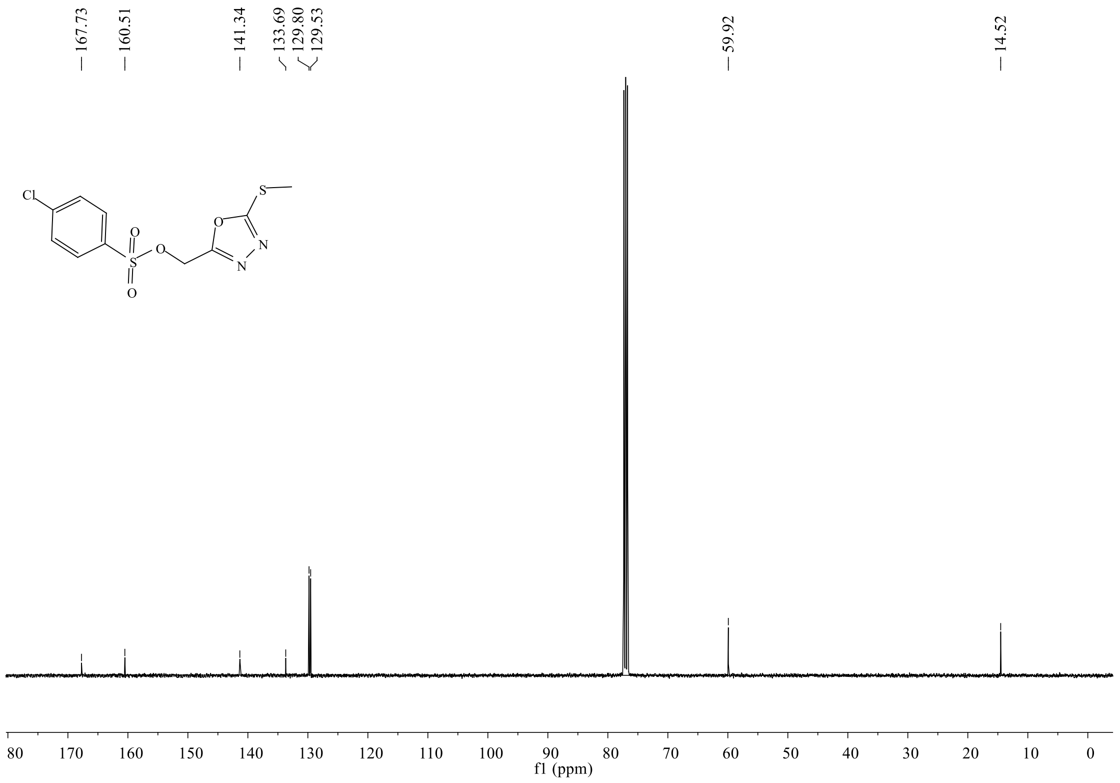

^{13}C NMR of compound **4a-3**

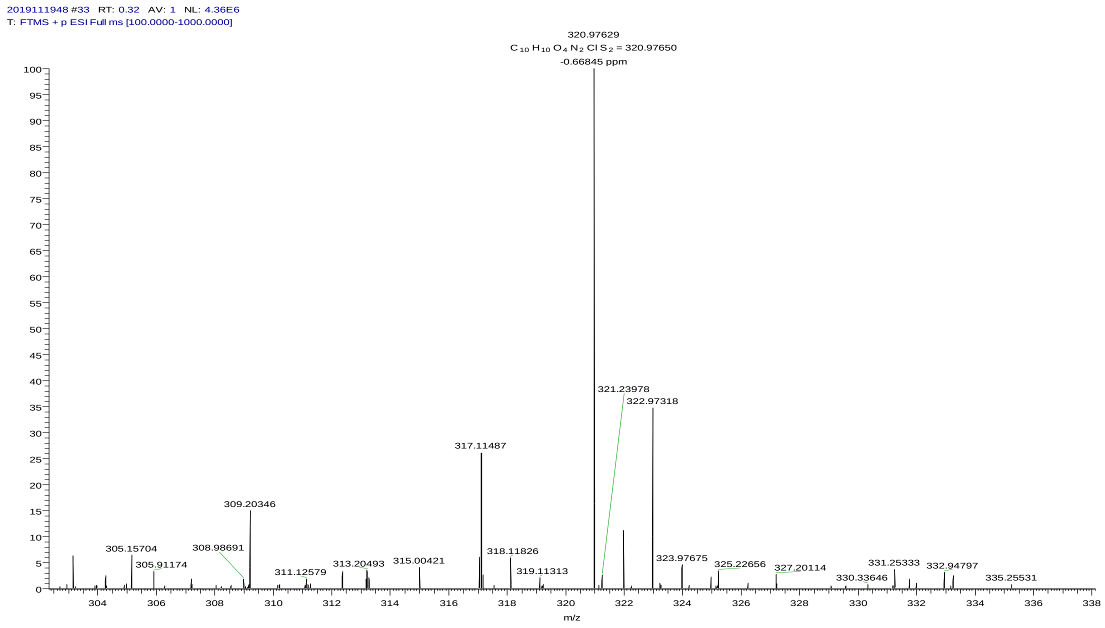

HRMS of compound **4a-3**

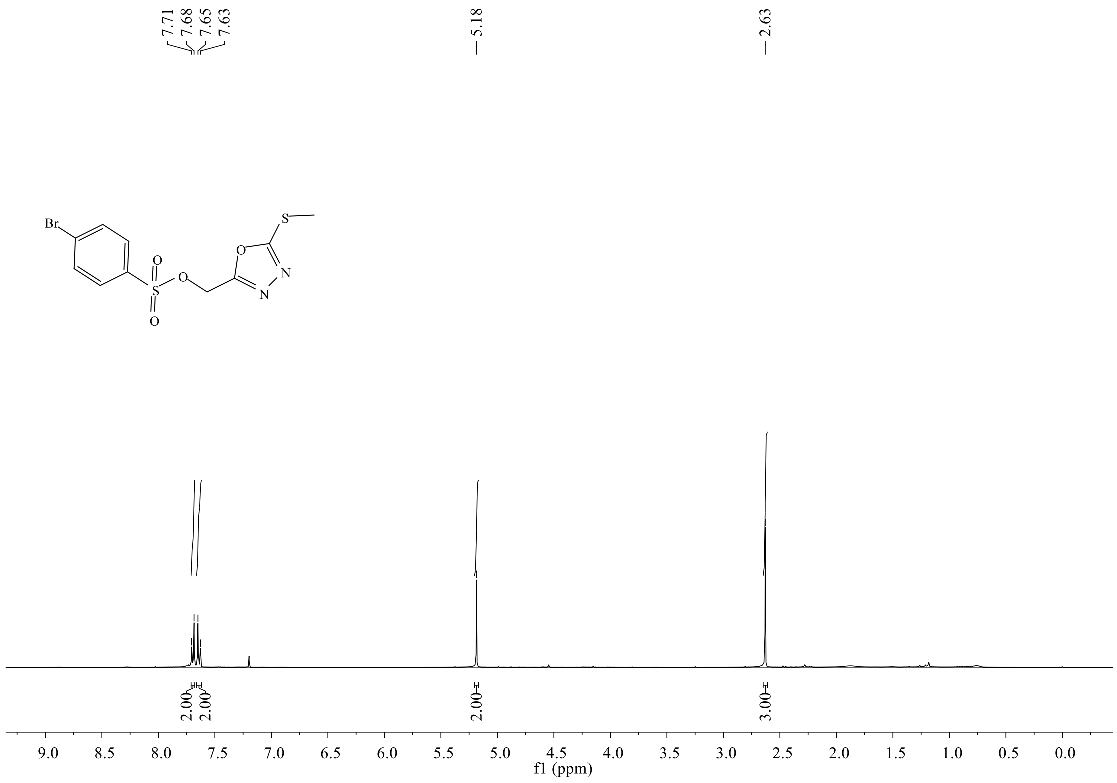

^{1}H NMR of compound **4a-4**

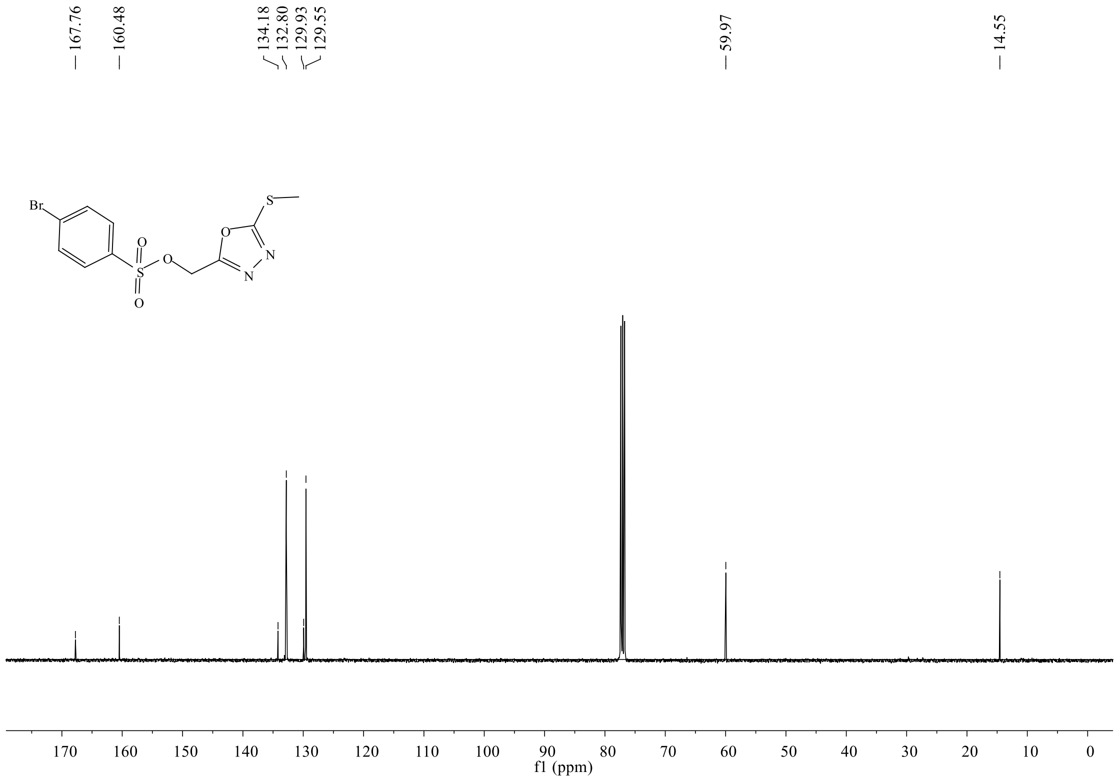

^{13}C NMR of compound **4a-4**

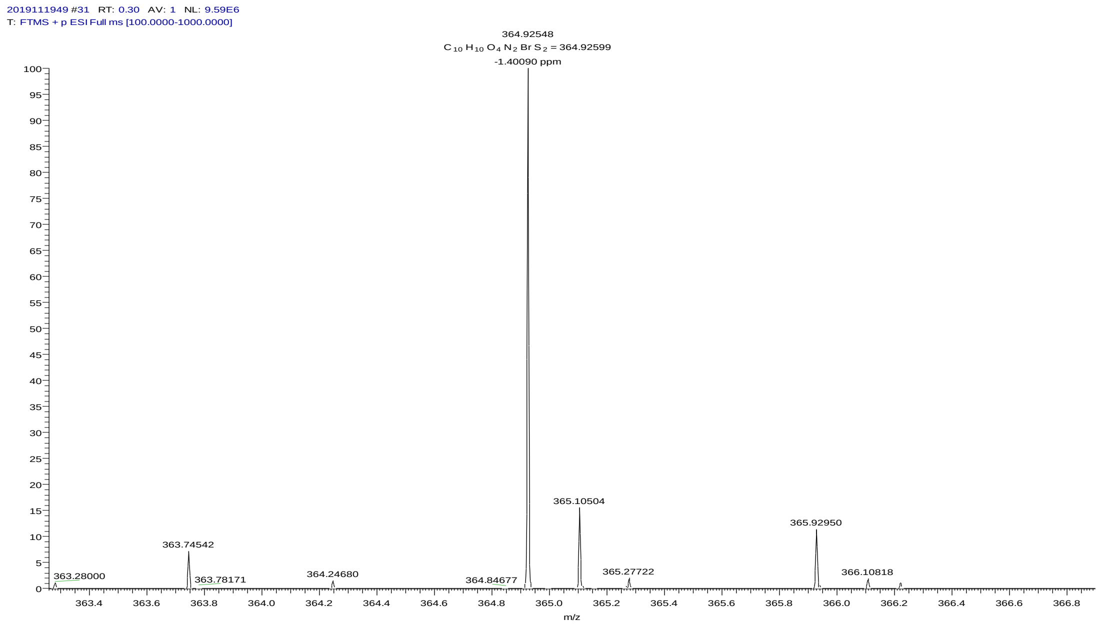

HRMS of compound **4a-4**

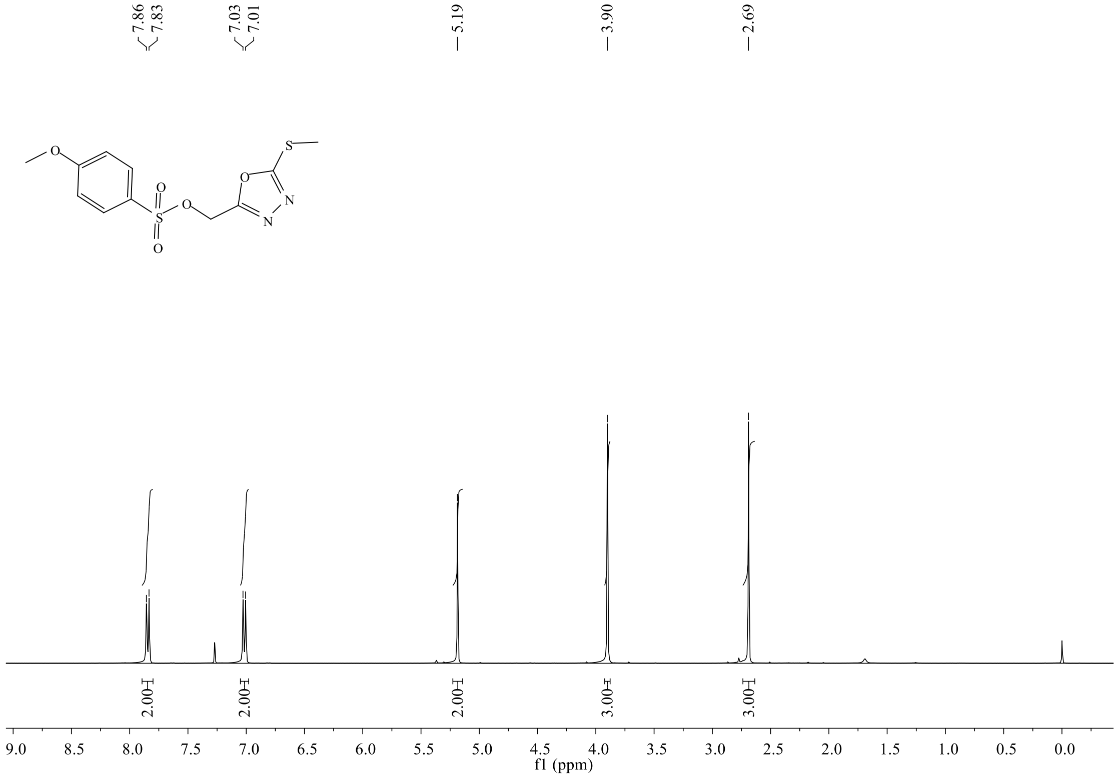

^{1}H NMR of compound **4a-5**

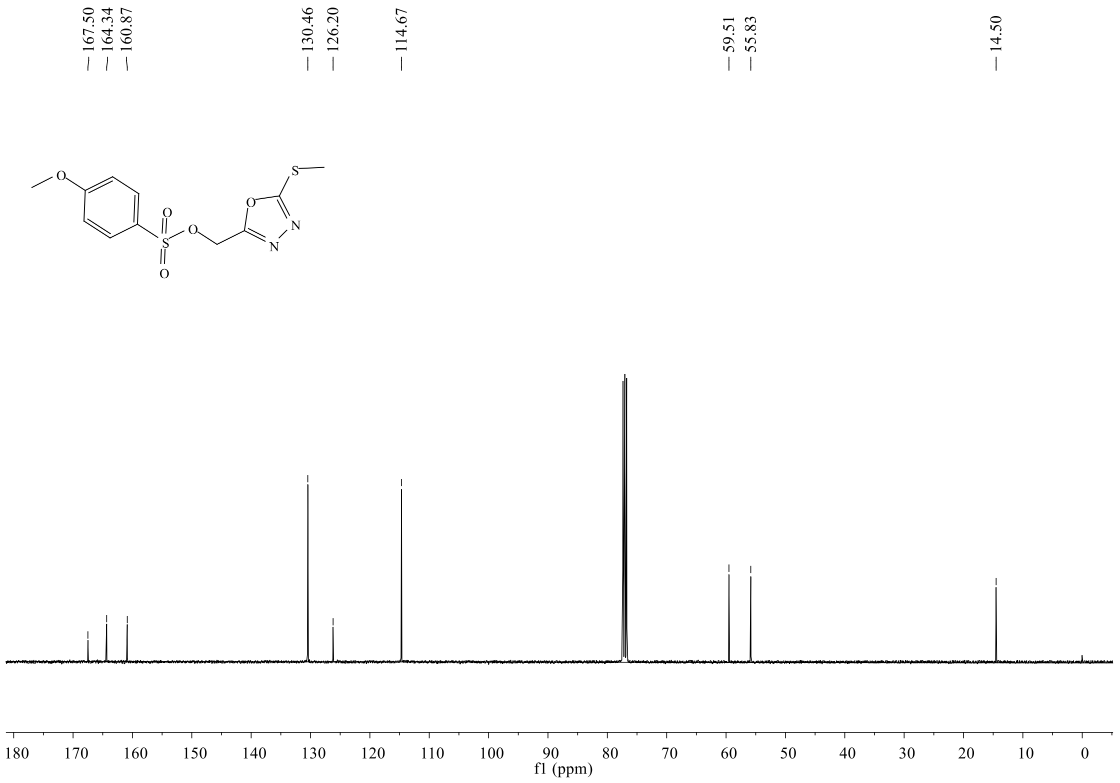

^{13}C NMR of compound **4a-5**

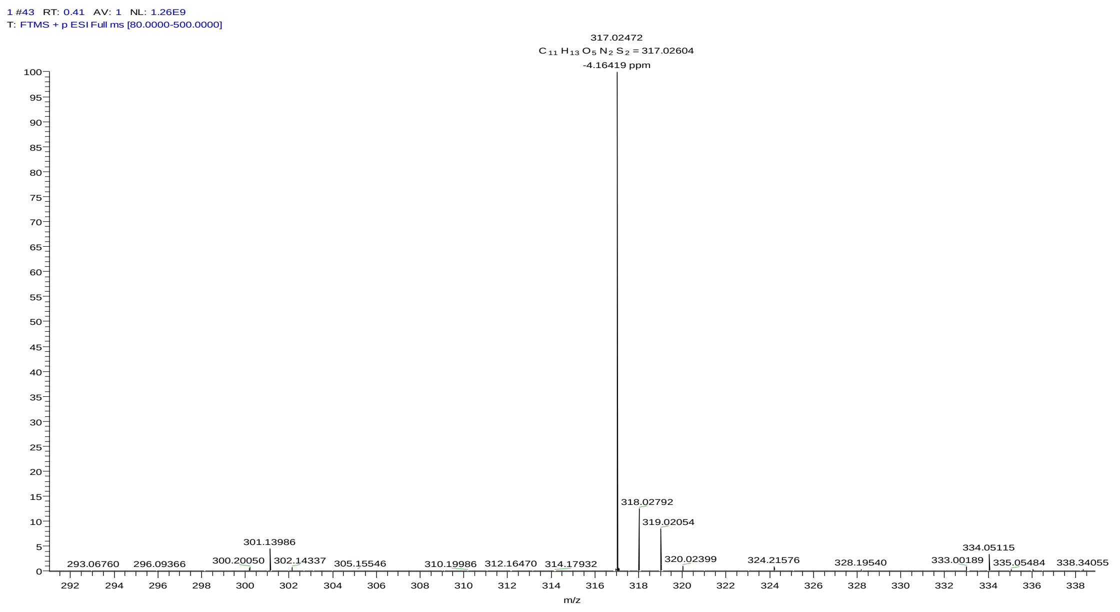

HRMS of compound **4a-5**

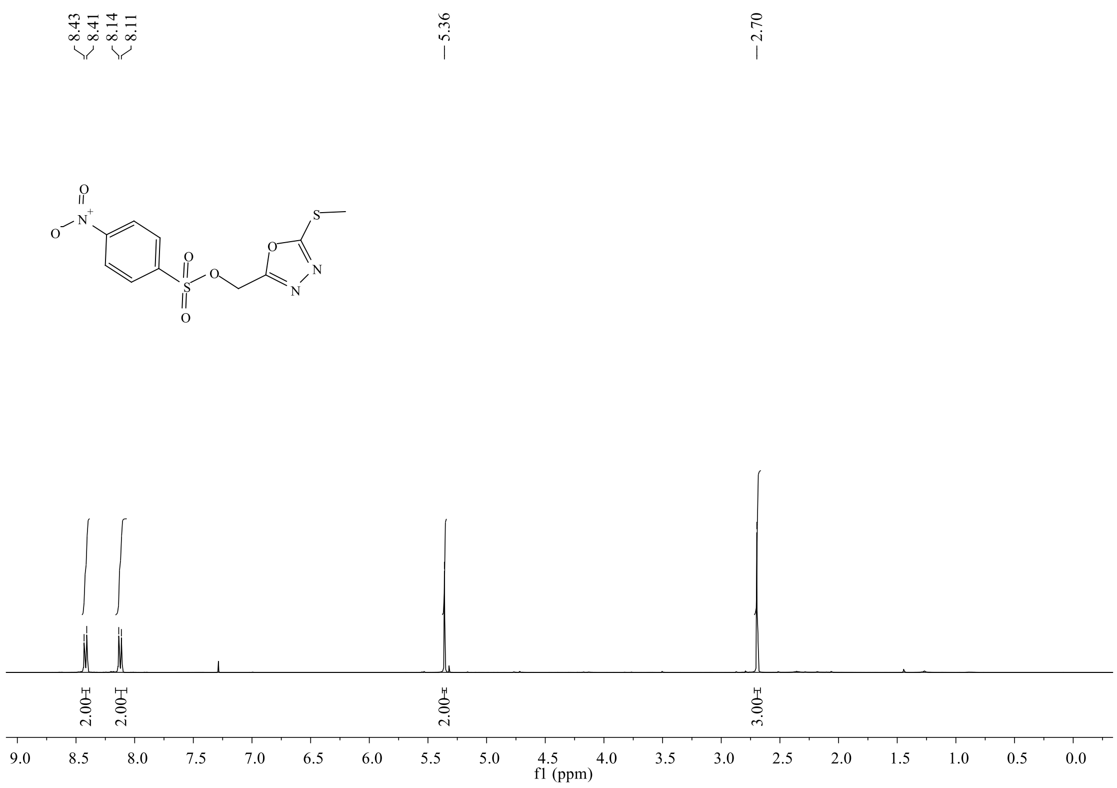

^{1}H NMR of compound **4a-6**

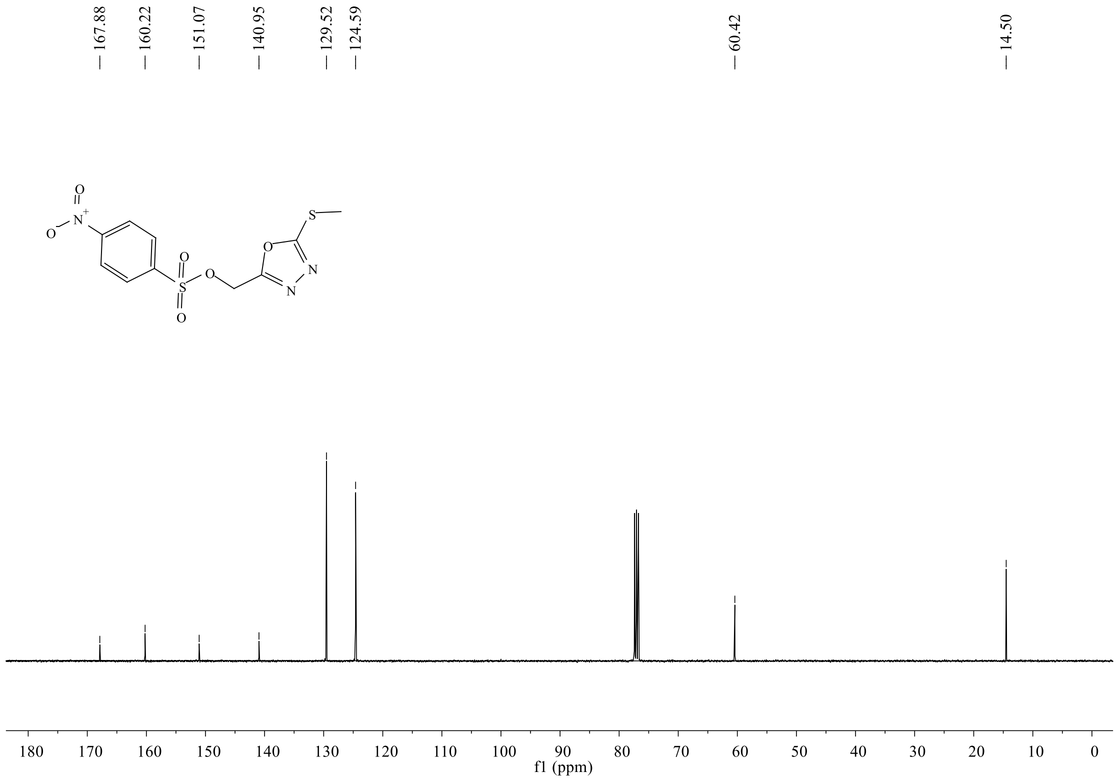

^{13}C NMR of compound **4a-6**

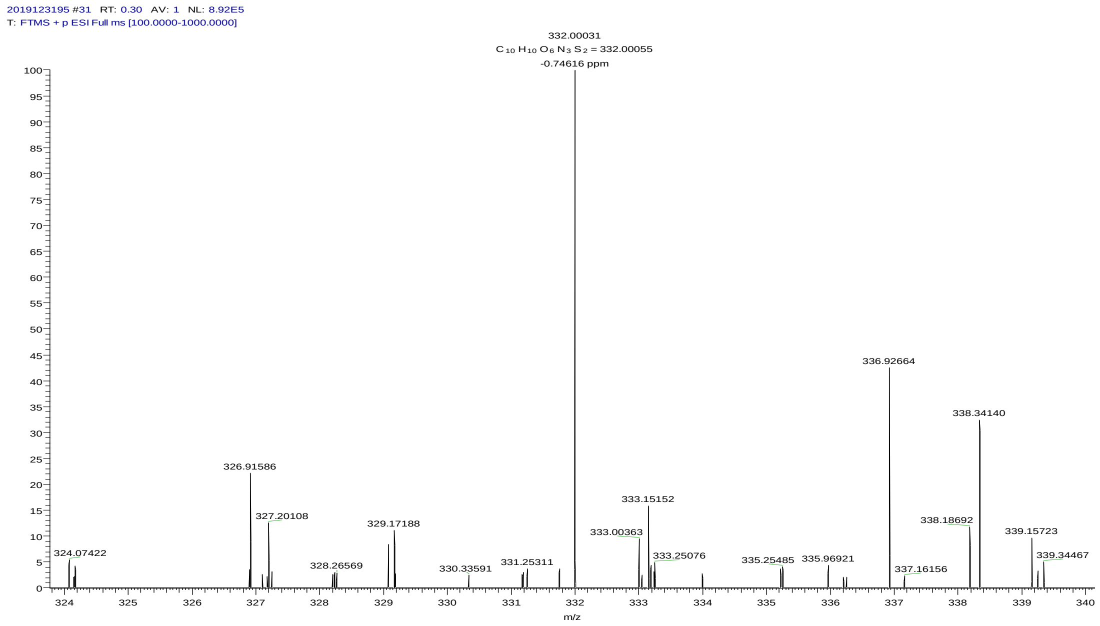

HRMS of compound **4a-6**

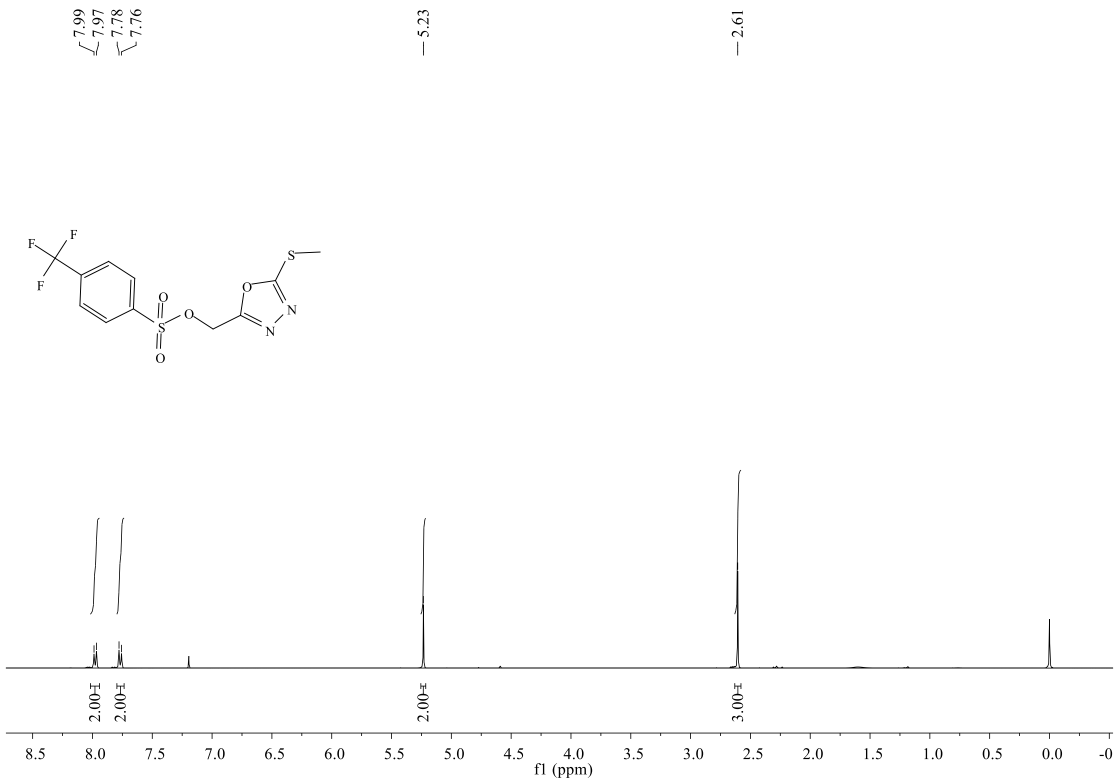

^{1}H NMR of compound **4a-7**

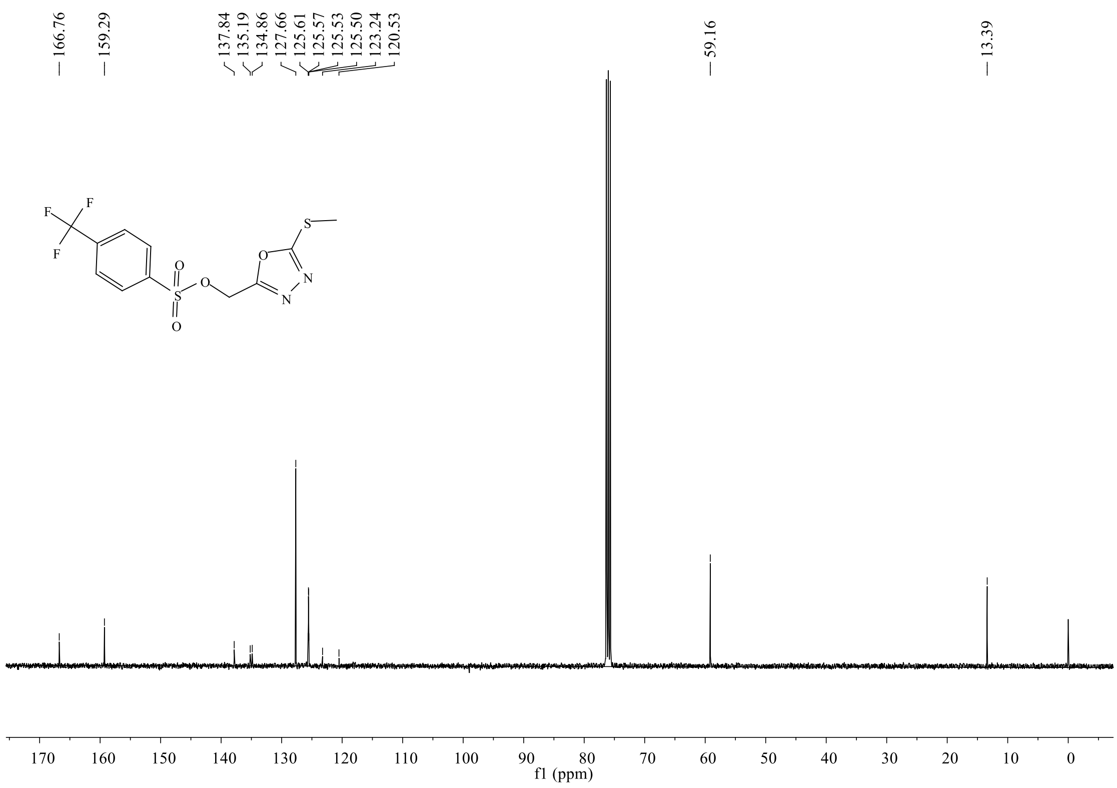

^{13}C NMR of compound **4a-7**

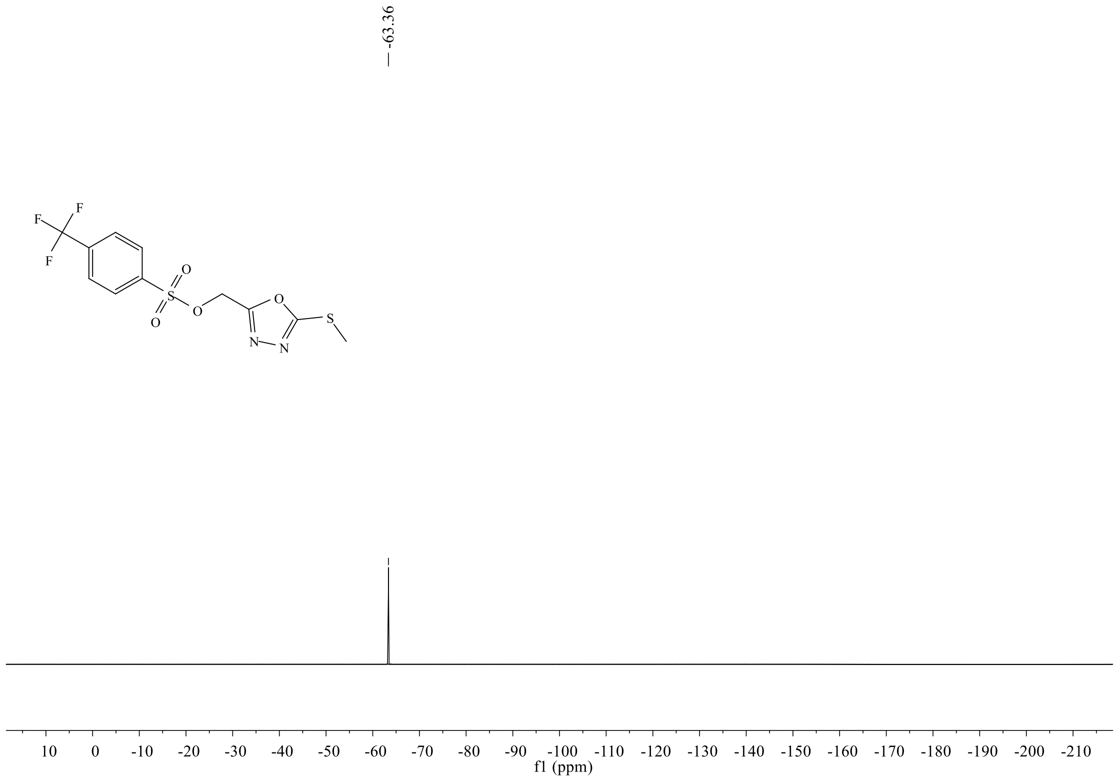

^{19}F NMR of compound **4a-7**

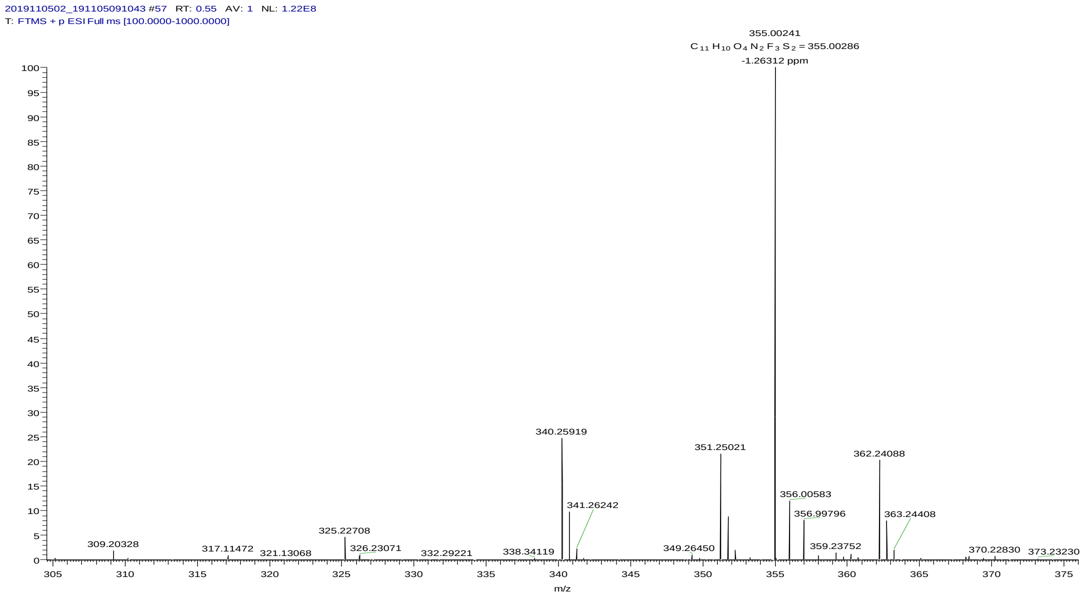

HRMS of compound **4a-7**

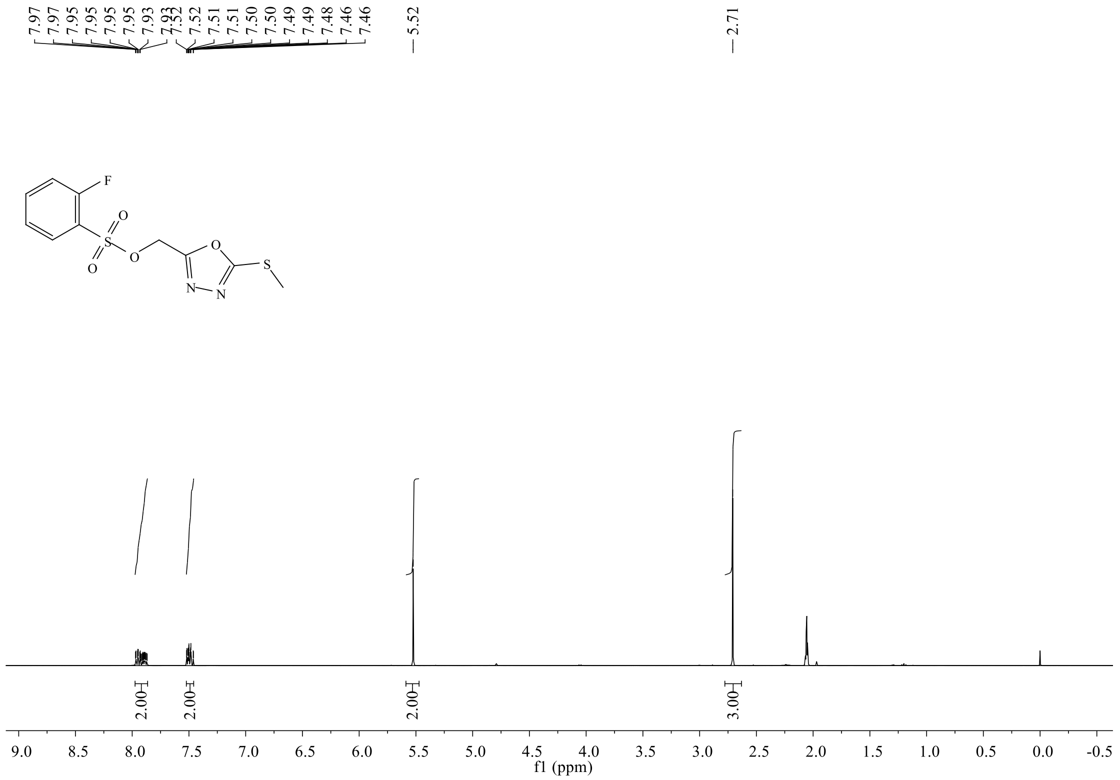

^{1}H NMR of compound **4a-8**

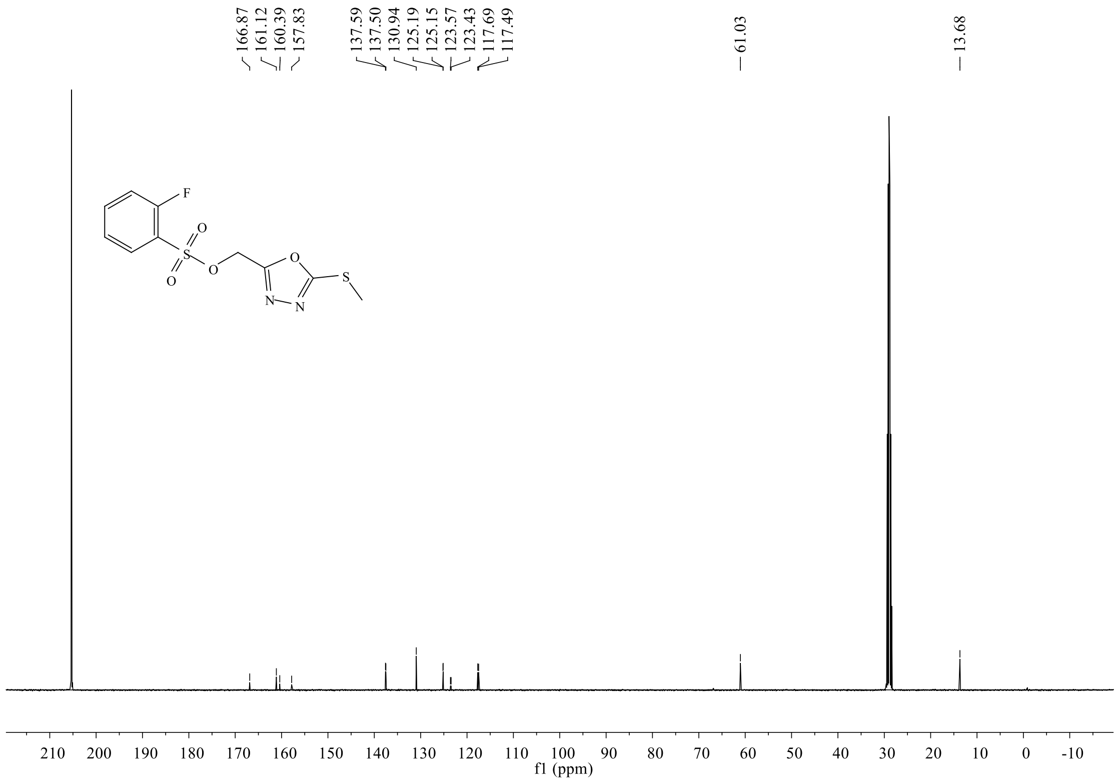

^{13}C NMR of compound **4a-8**

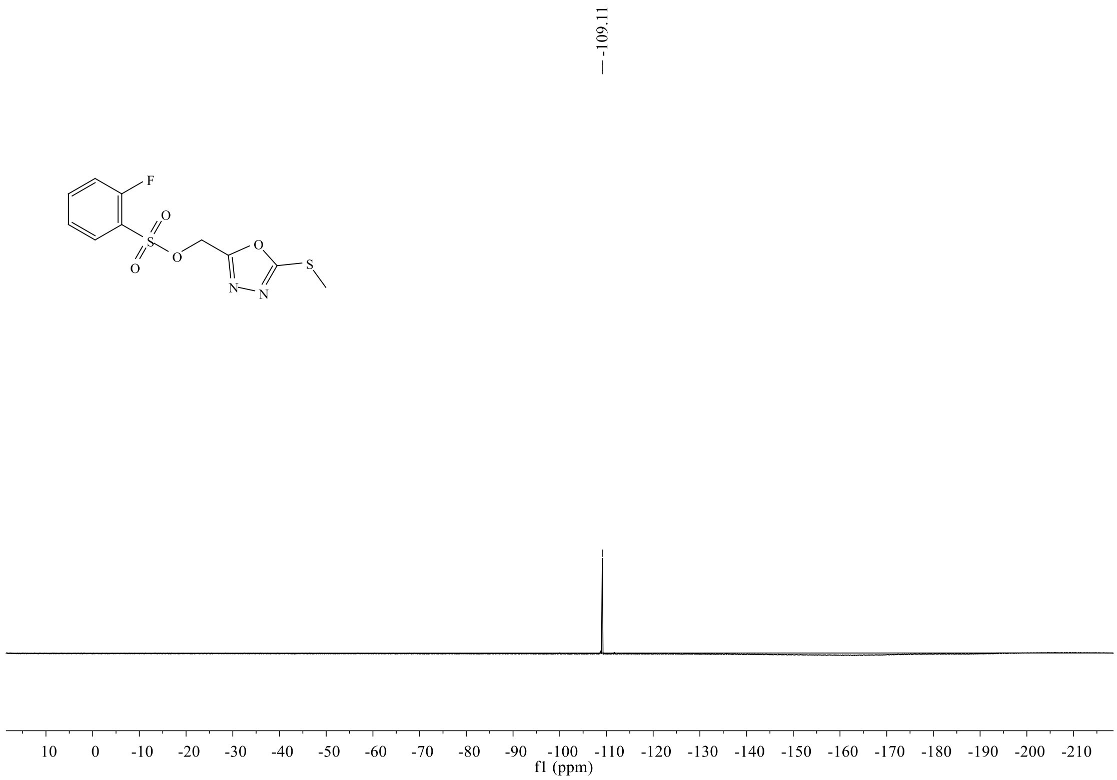

^{19}F NMR of compound **4a-8**

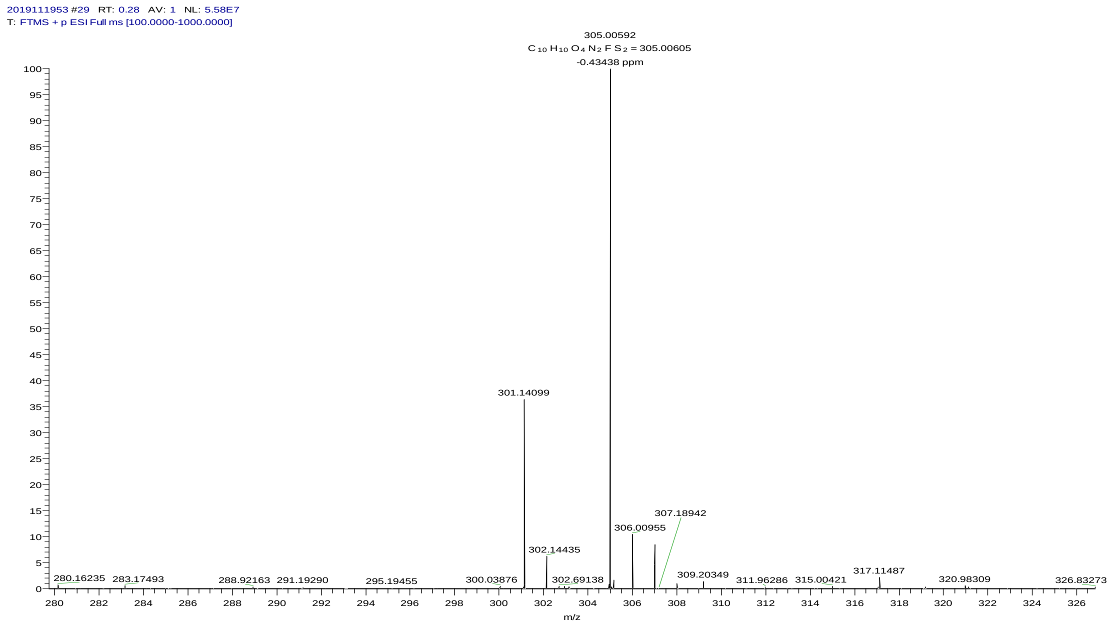

HRMS of compound **4a-8**

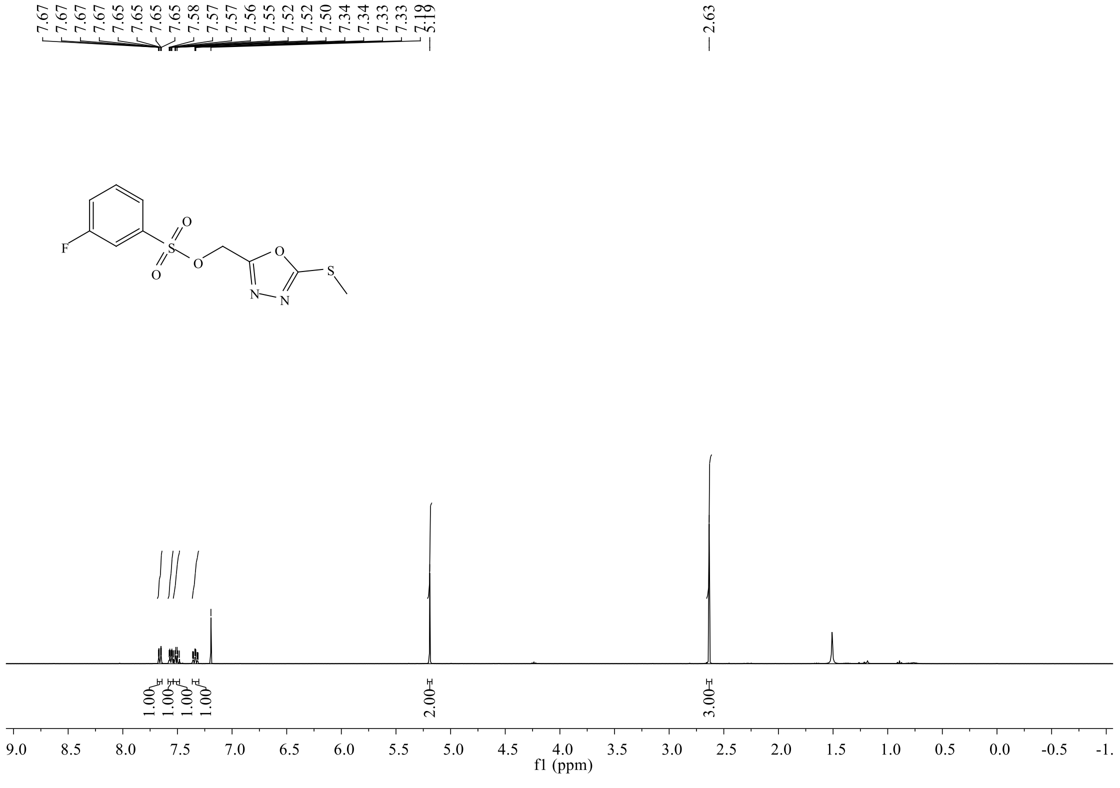

^{1}H NMR of compound **4a-9**

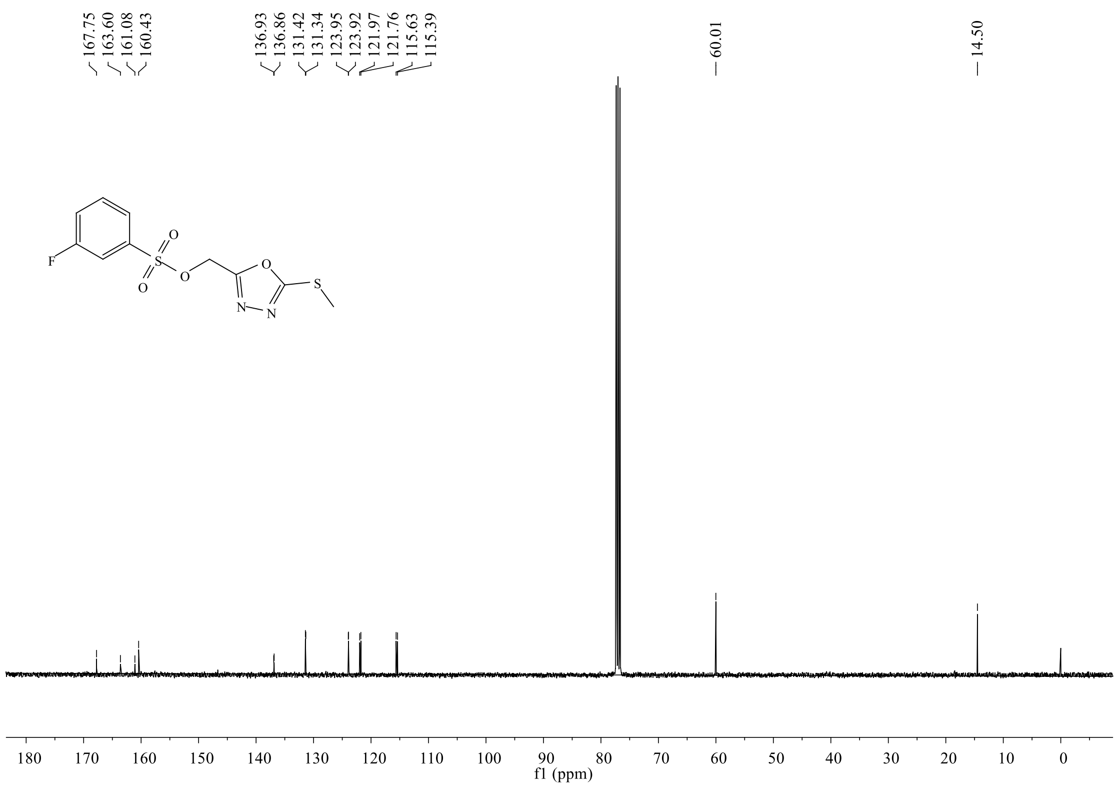

^{13}C NMR of compound **4a-9**

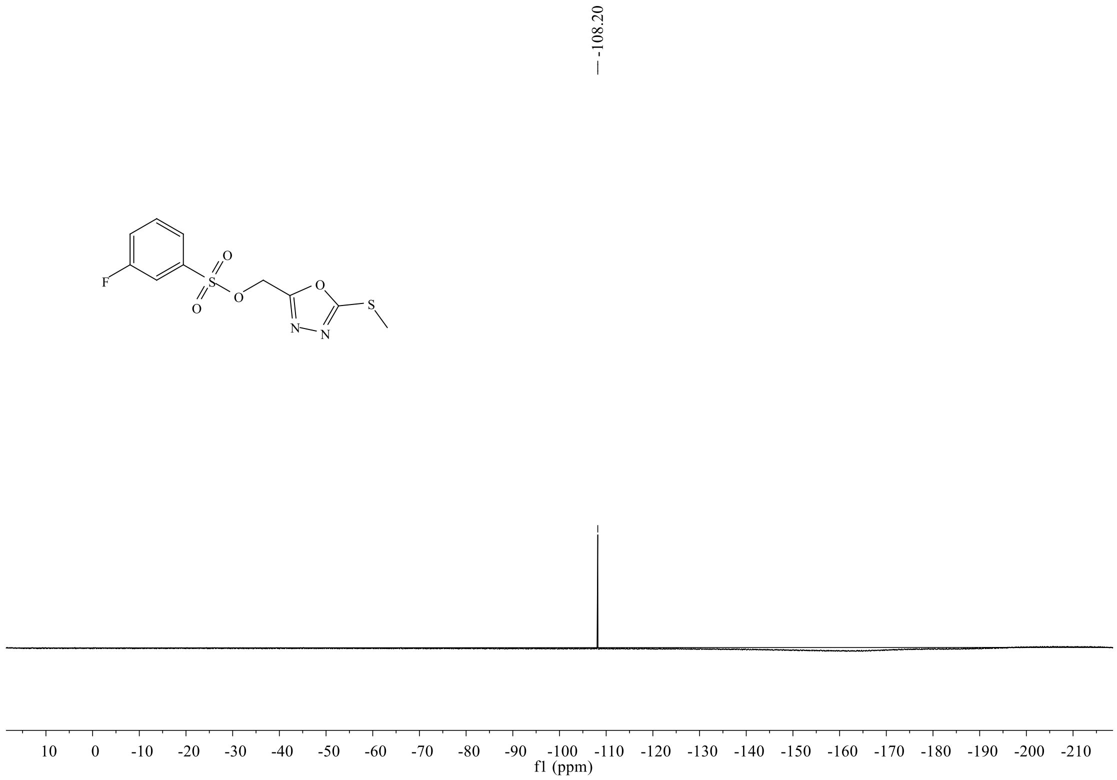

^{19}F NMR of compound **4a-9**

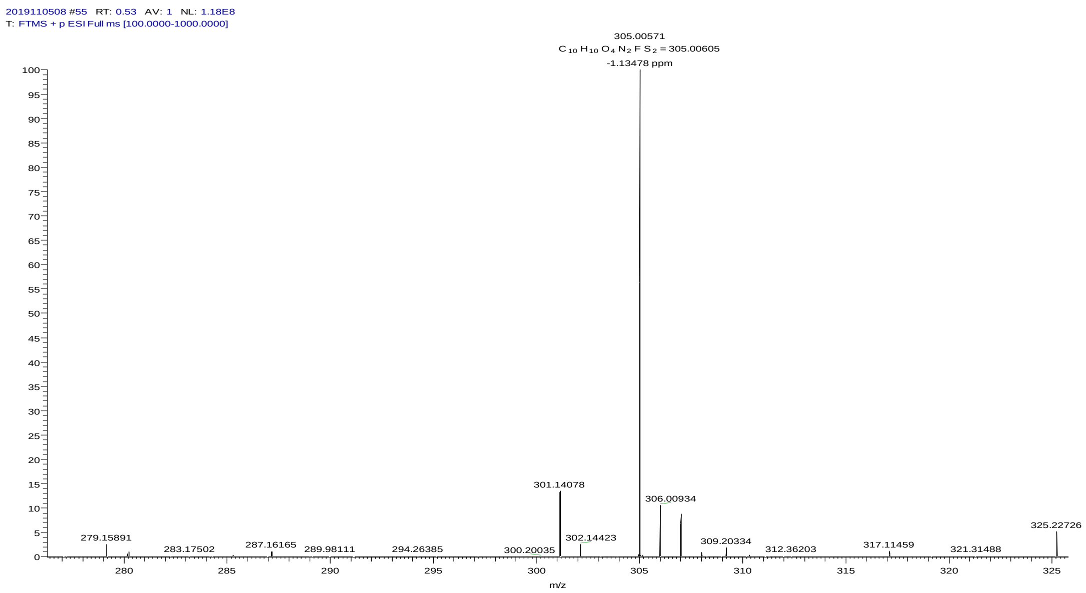

HRMS of compound **4a-9**

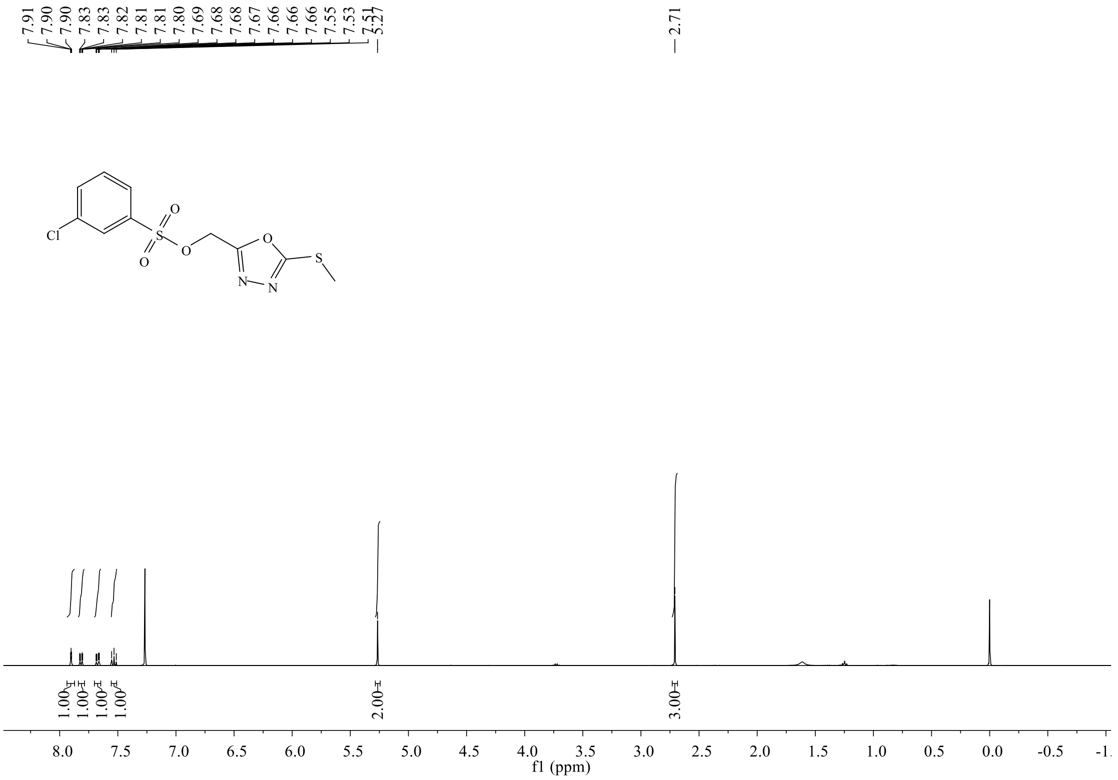

^{1}H NMR of compound **4a-10**

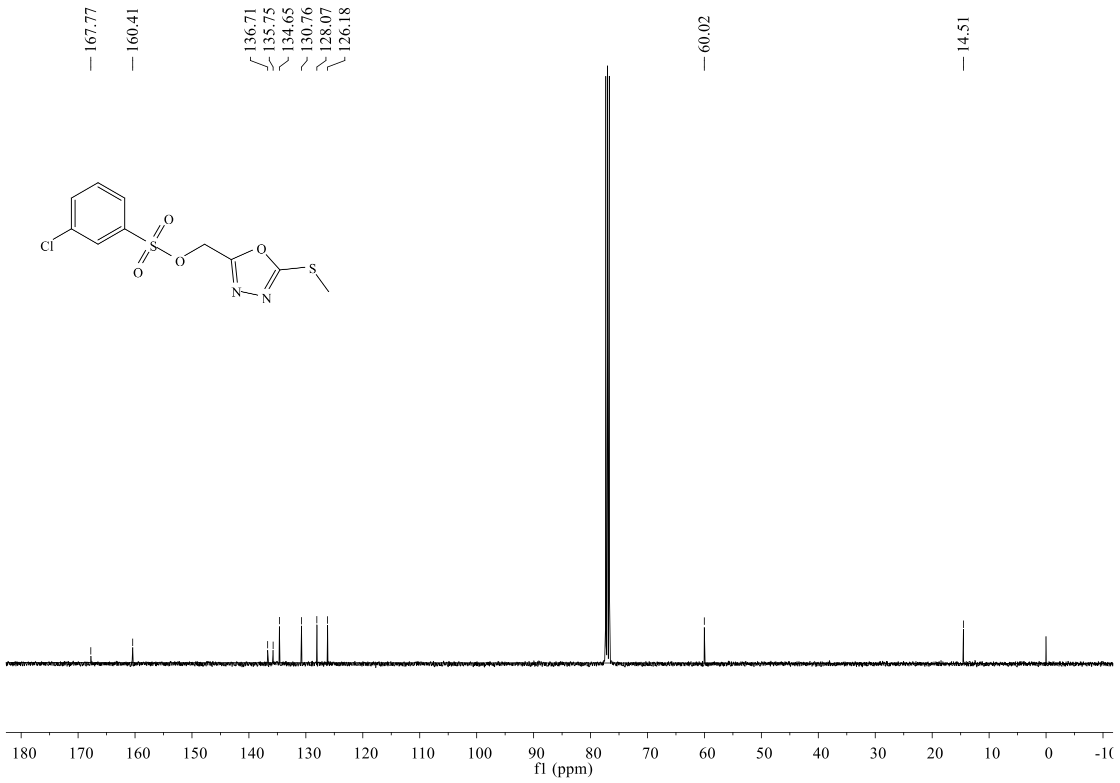

^{13}C NMR of compound **4a-10**

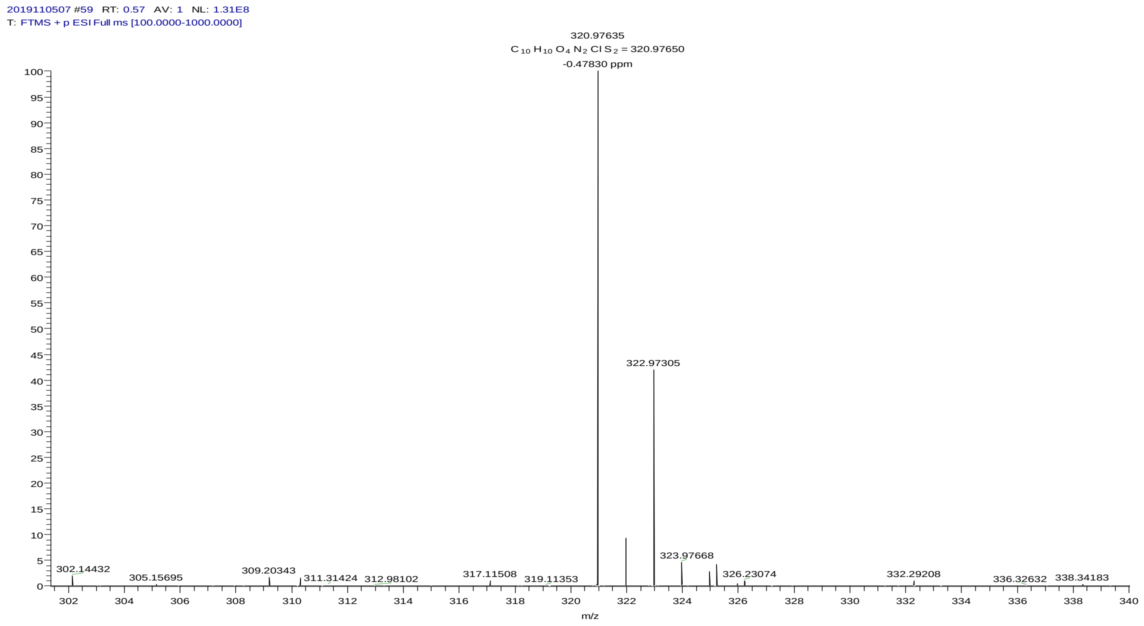

HRMS of compound **4a-10**

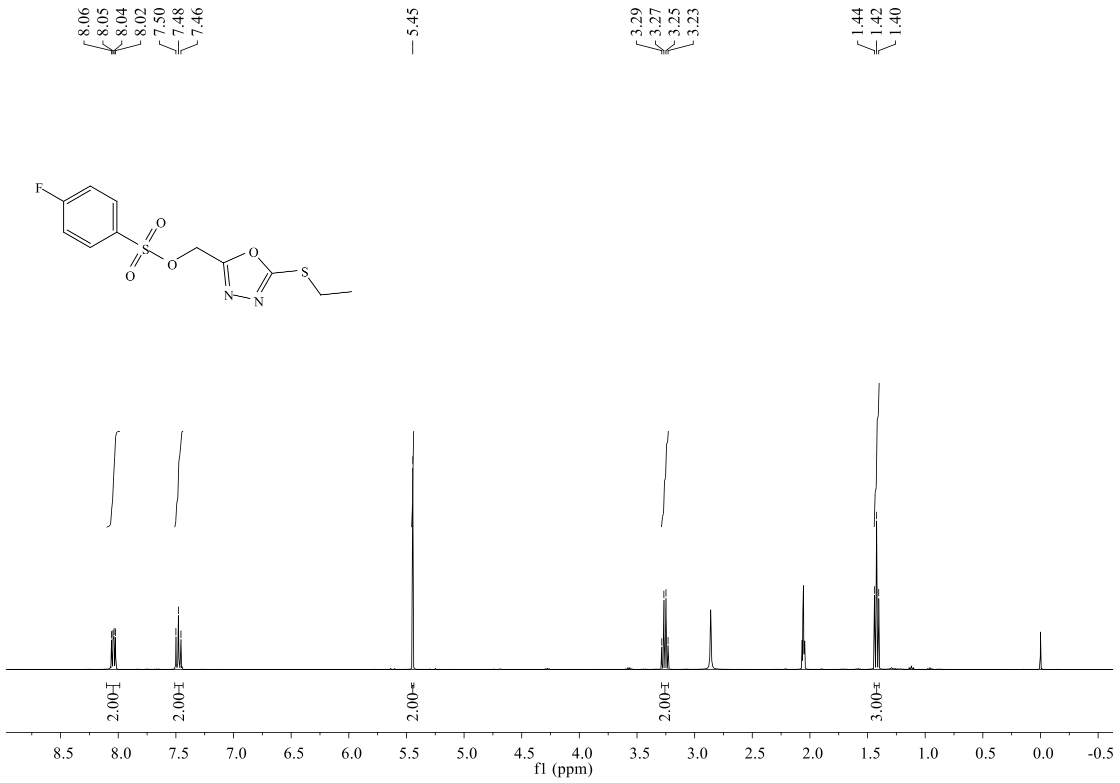

^{1}H NMR of compound **4a-11**

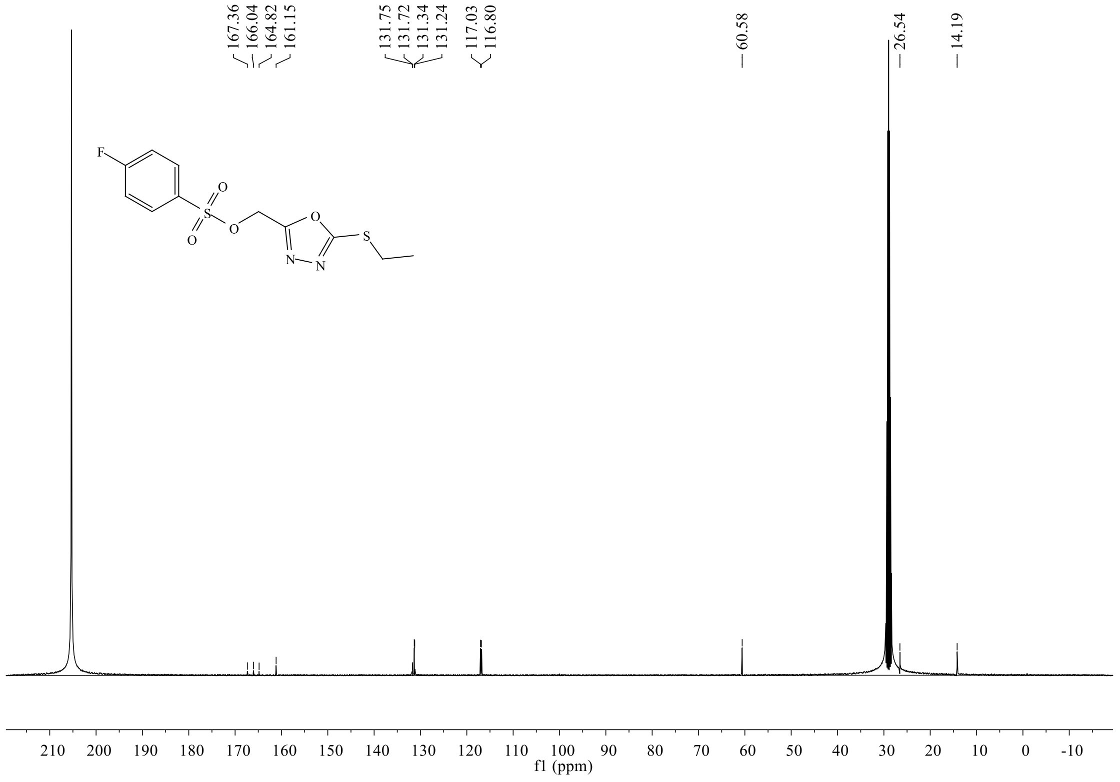

^{13}C NMR of compound **4a-11**

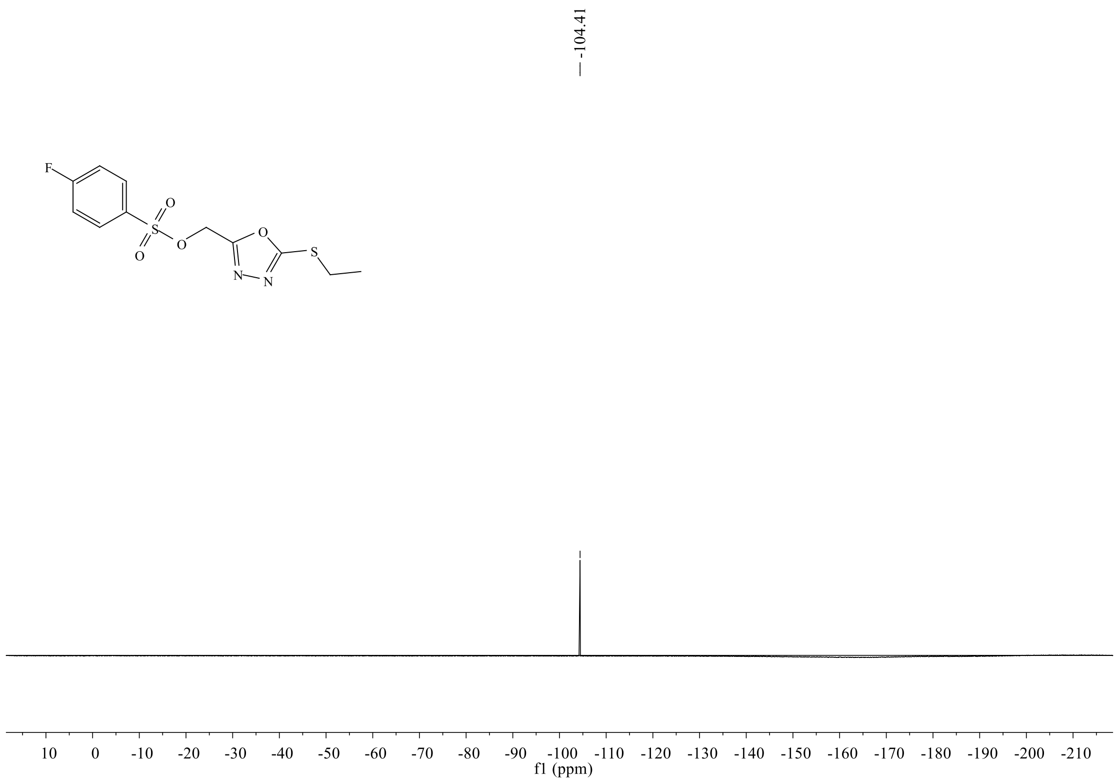

^{19}F NMR of compound **4a-11**

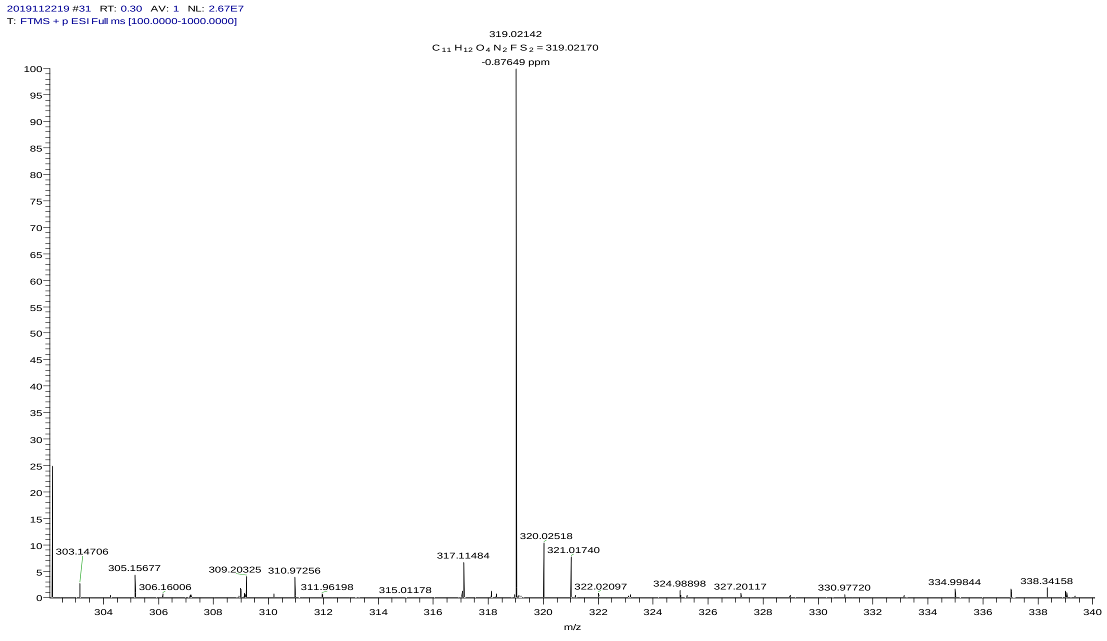

HRMS of compound **4a-11**

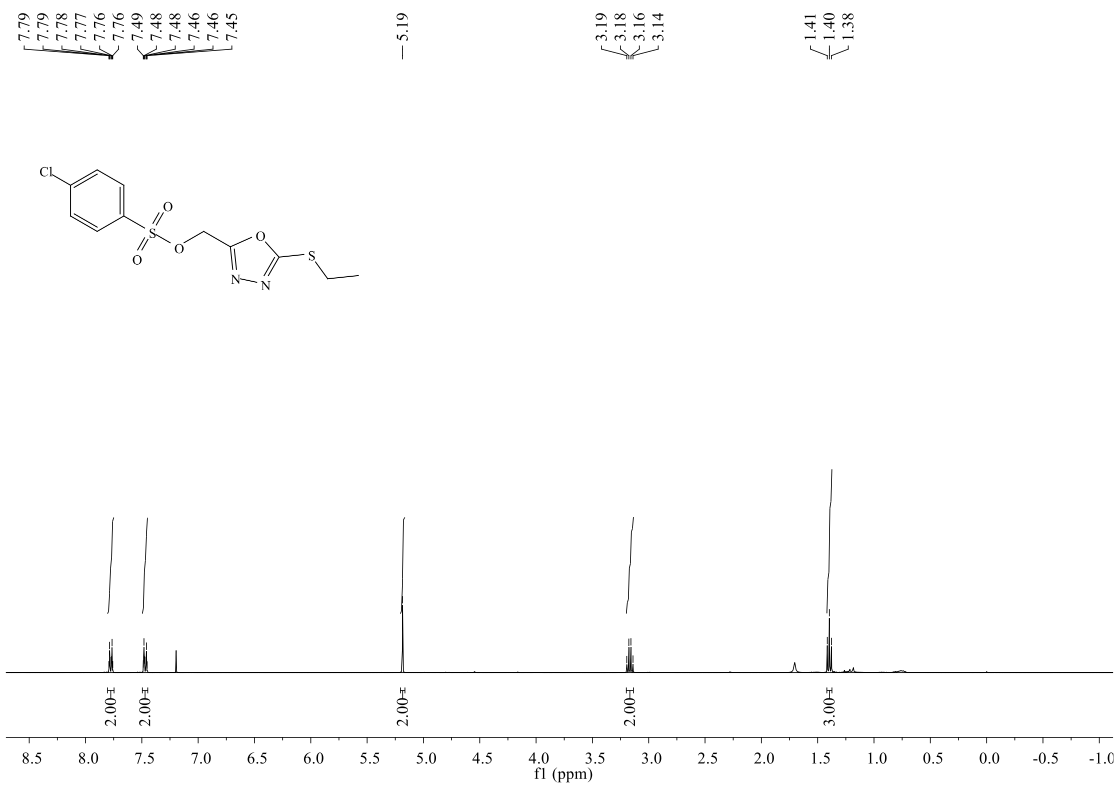

^{1}H NMR of compound **4a-12**

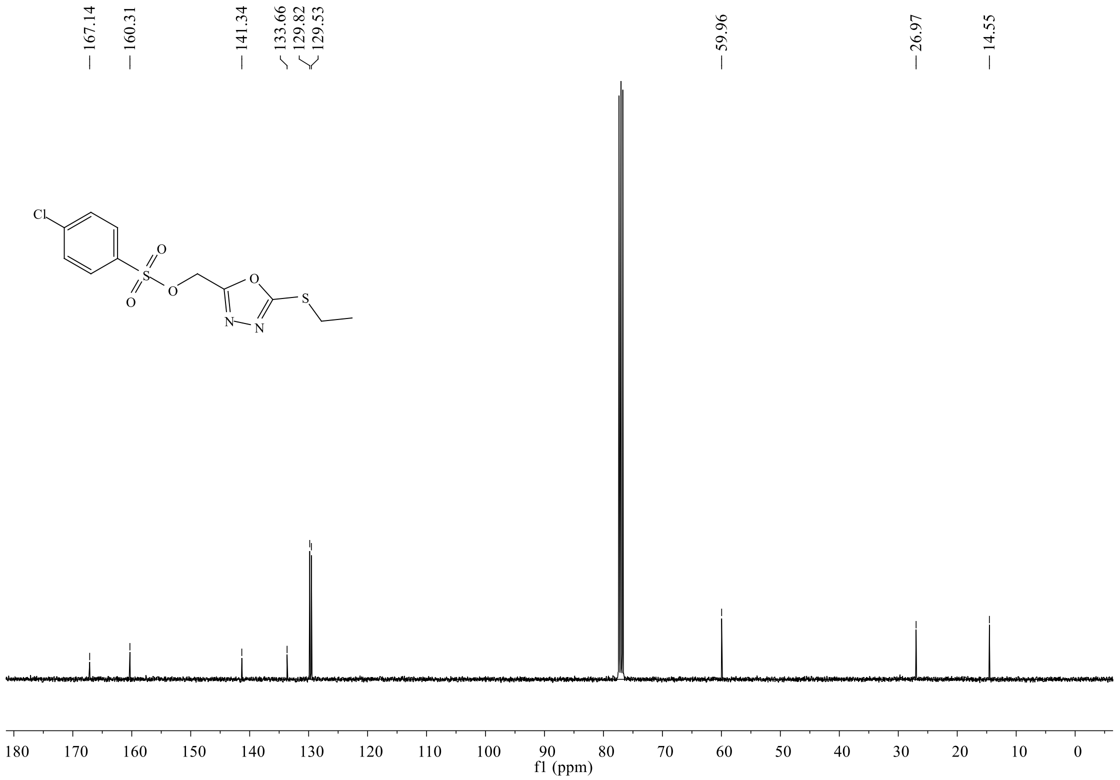

^{13}C NMR of compound **4a-12**

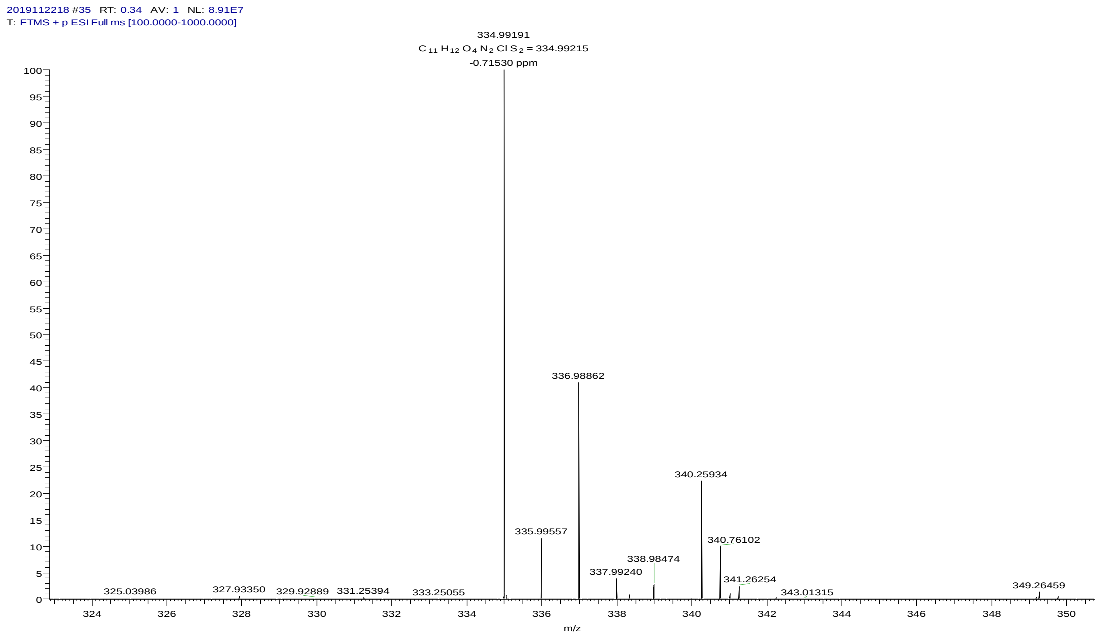

HRMS of compound **4a-12**

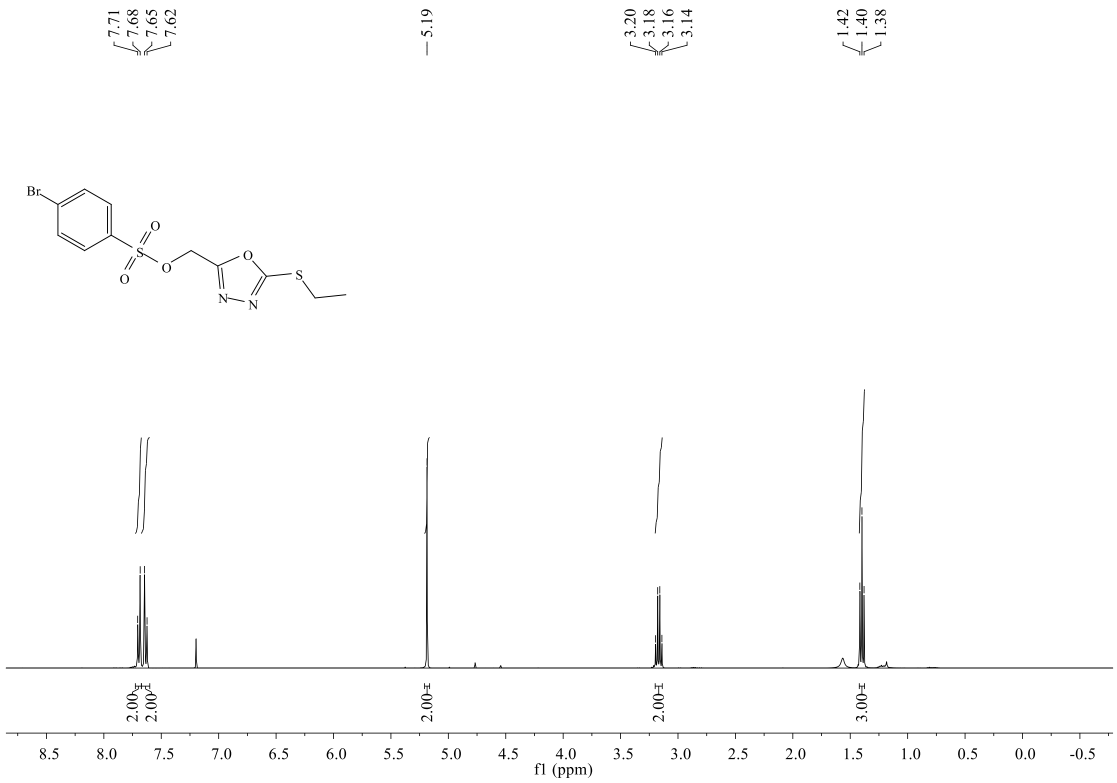

^{1}H NMR of compound **4a-13**

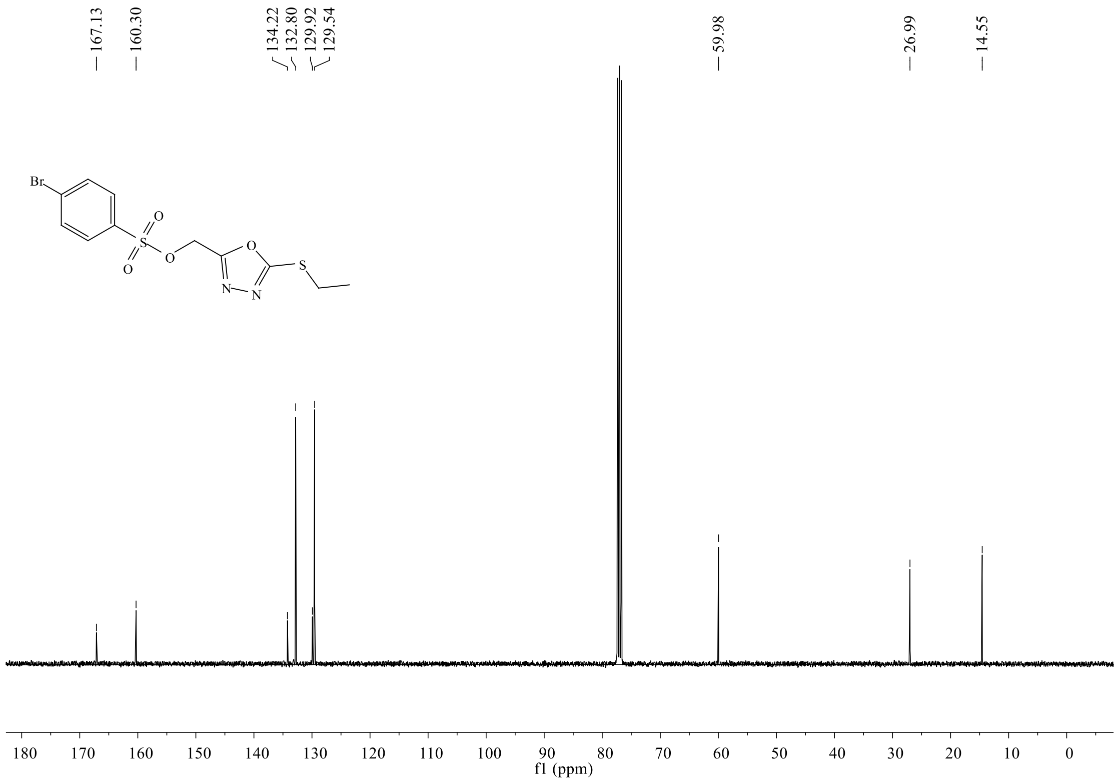

^{13}C NMR of compound **4a-13**

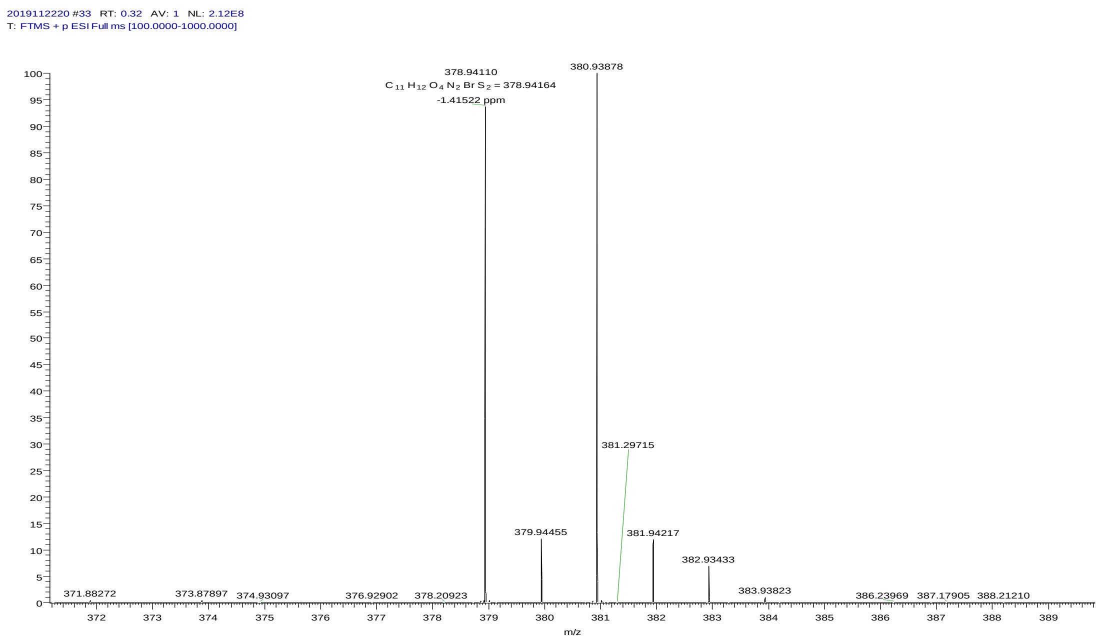

HRMS of compound **4a-13**

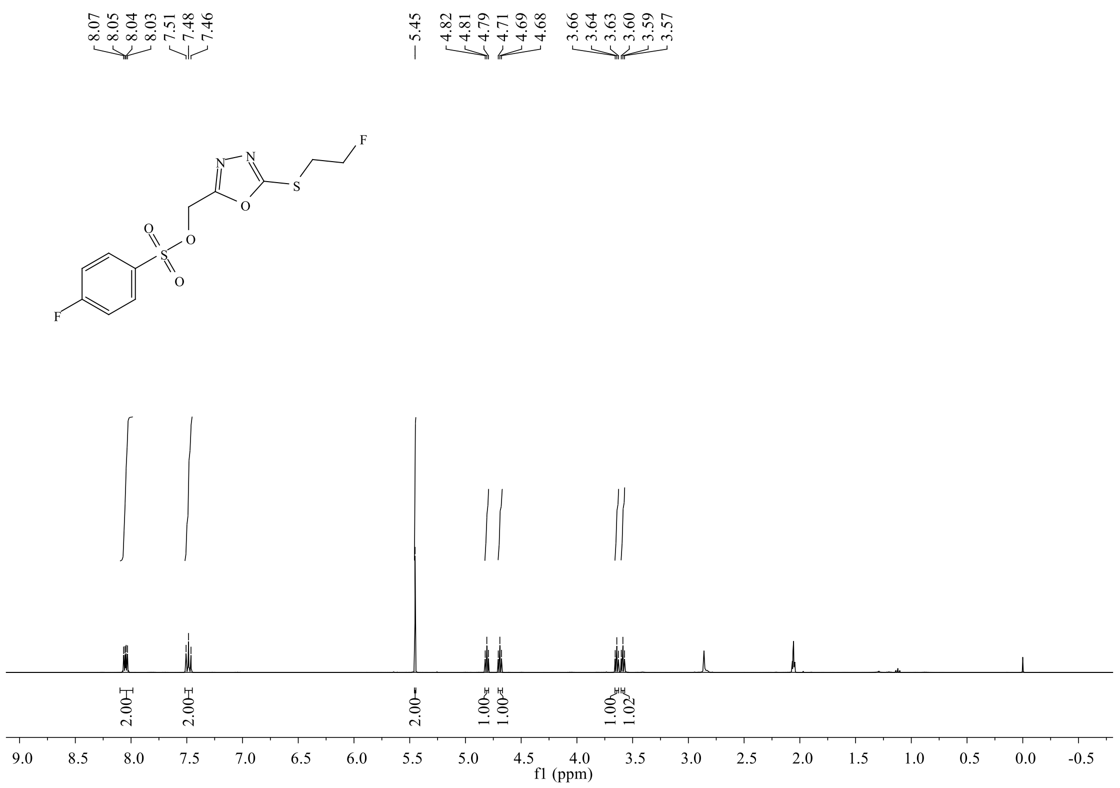

^{1}H NMR of compound **4a-14**

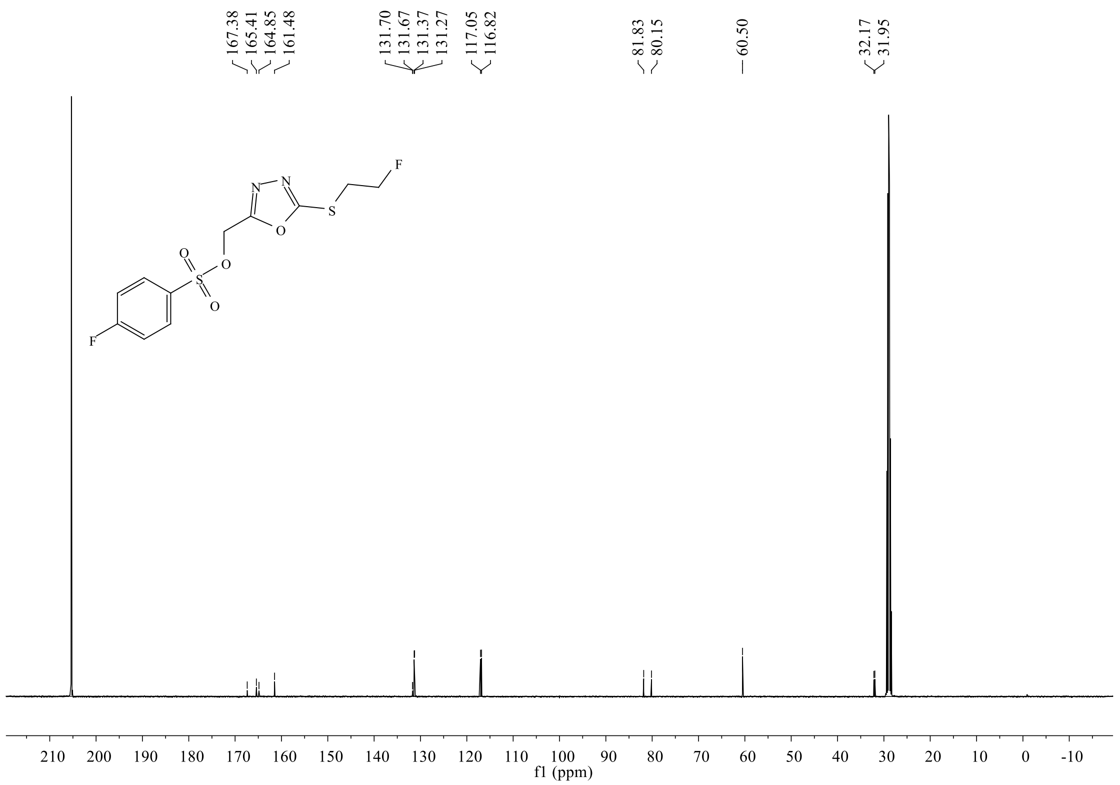

^{13}C NMR of compound **4a-14**

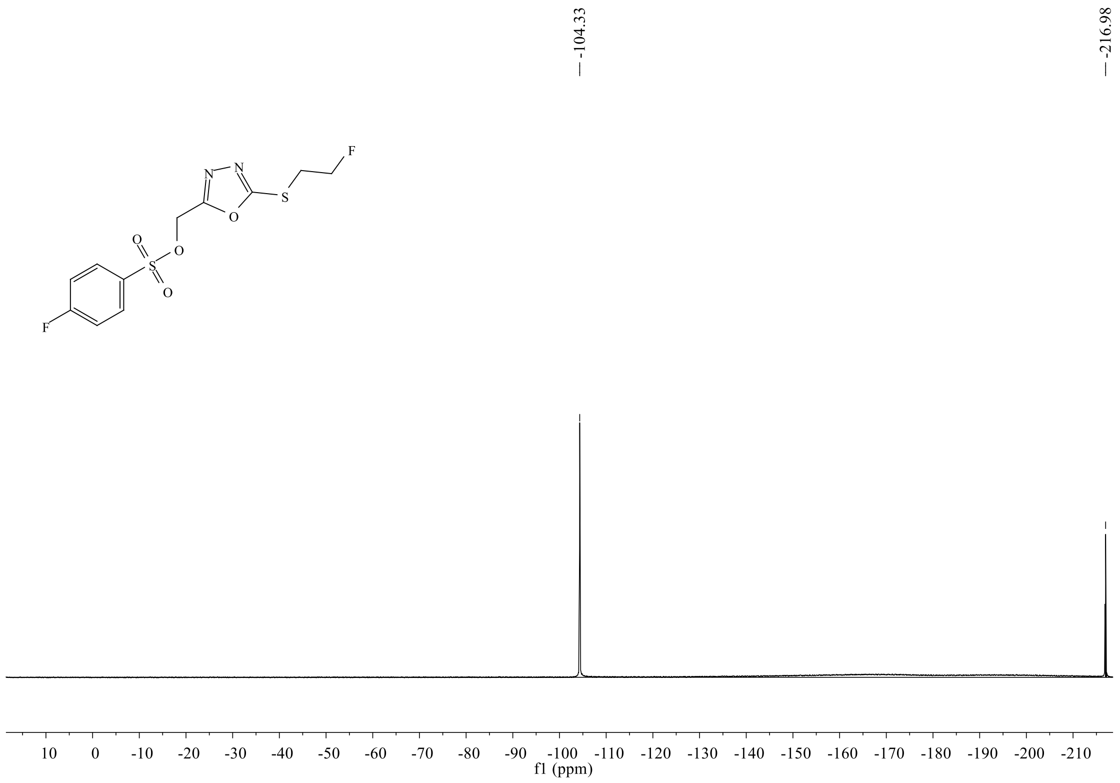

^{19}F NMR of compound **4a-14**

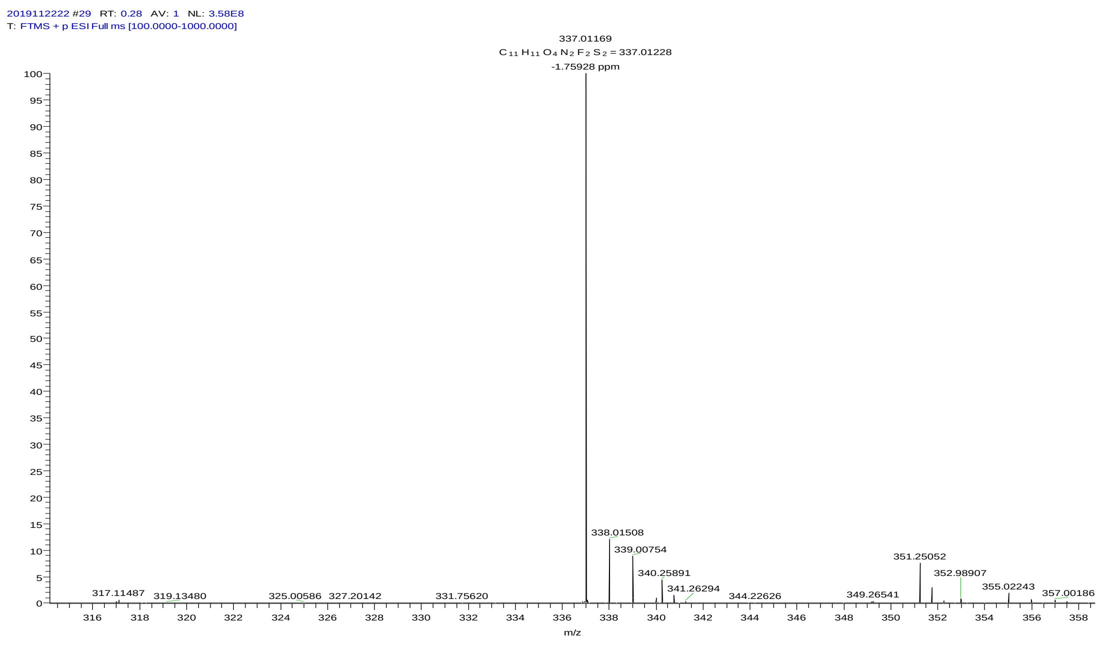

HRMS of compound **4a-14**

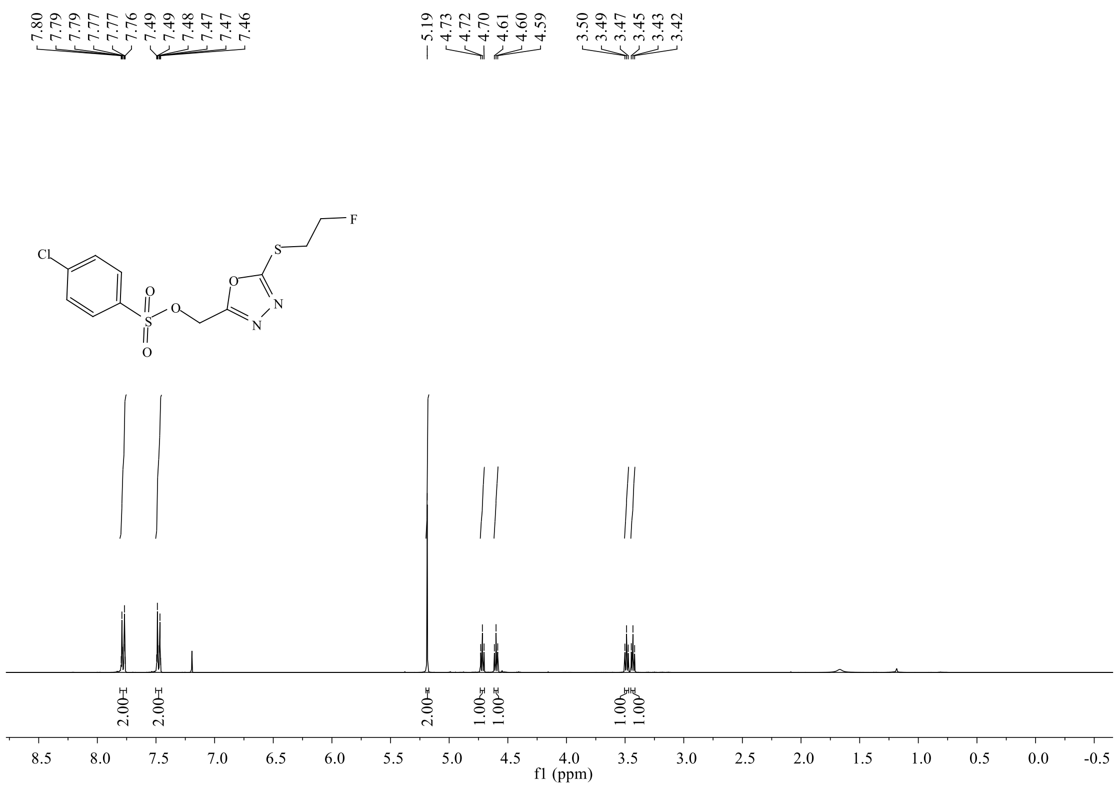

^{1}H NMR of compound **4a-15**

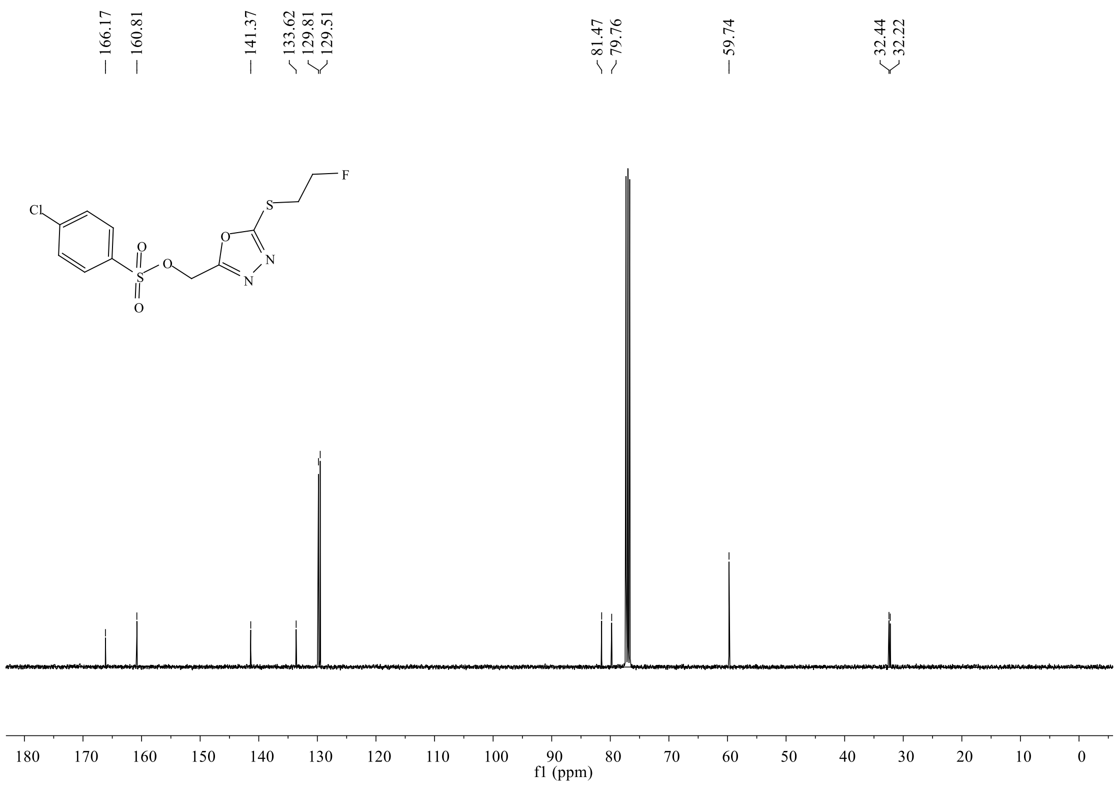

^{13}C NMR of compound **4a-15**

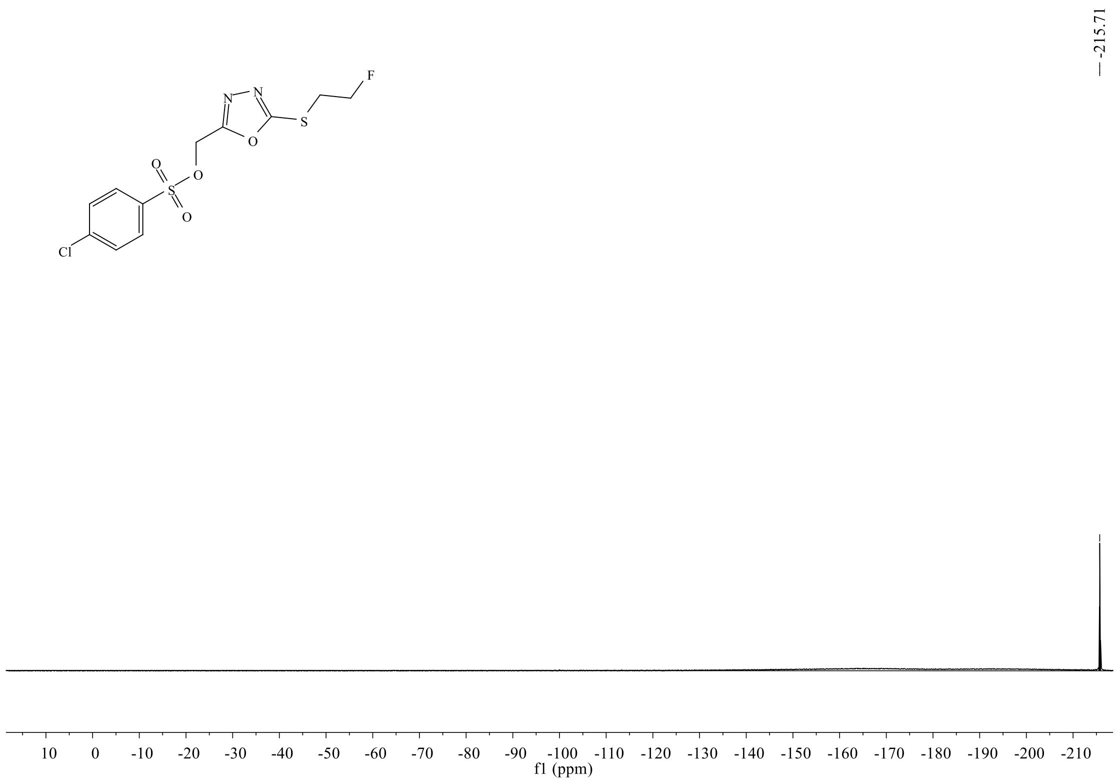

^{19}F NMR of compound **4a-15**

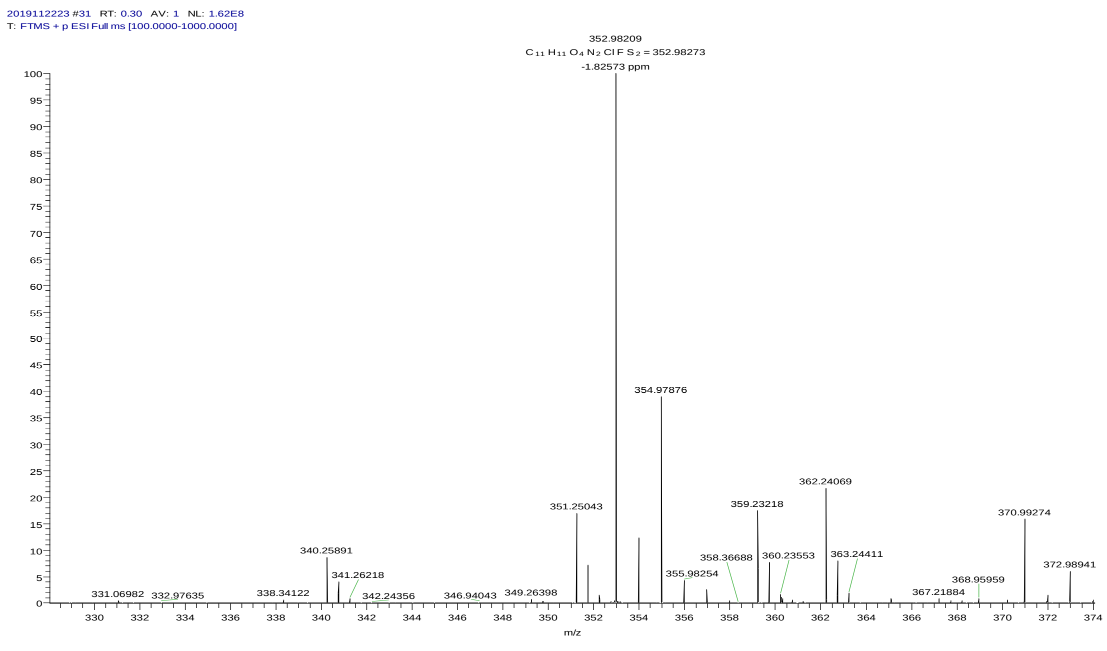

HRMS of compound **4a-15**

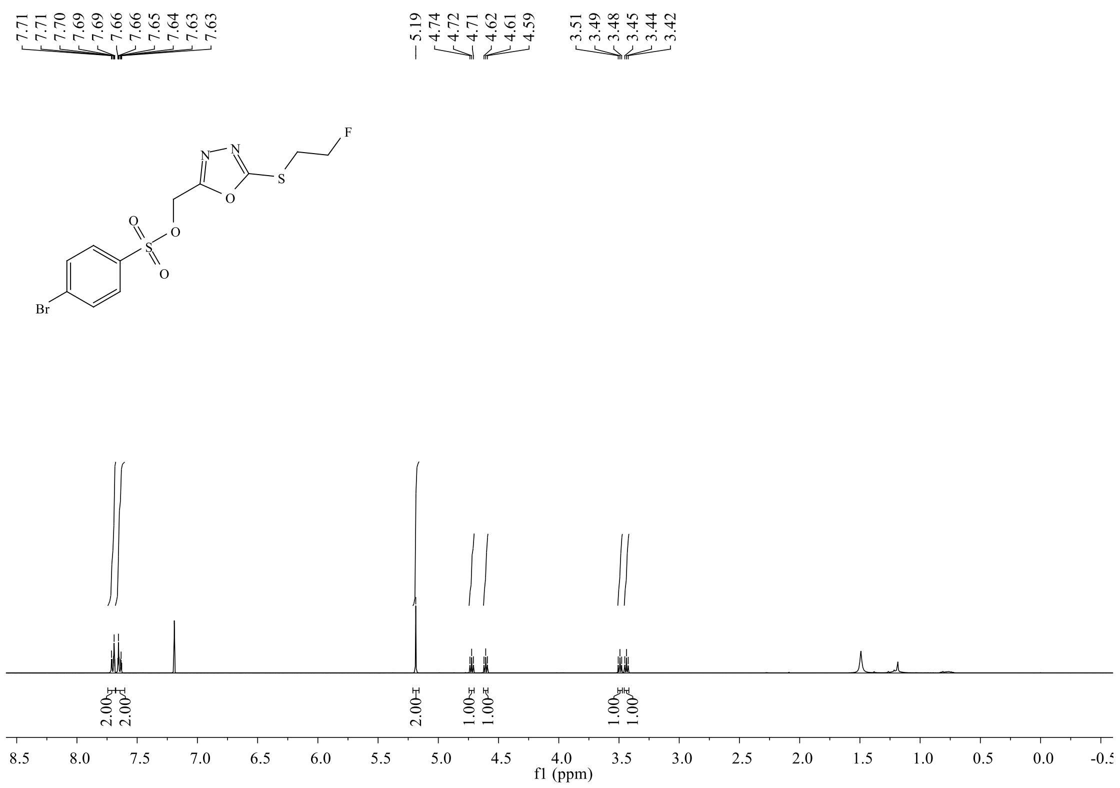

^{1}H NMR of compound **4a-16**

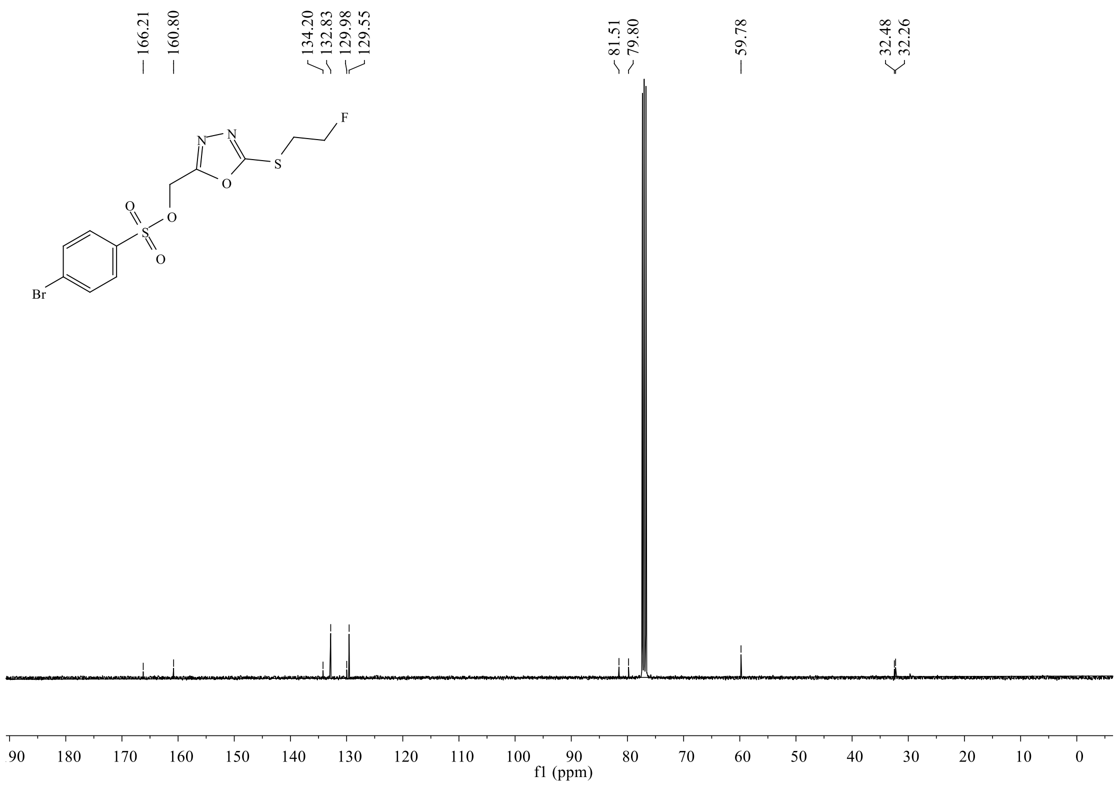

^{13}C NMR of compound **4a-16**

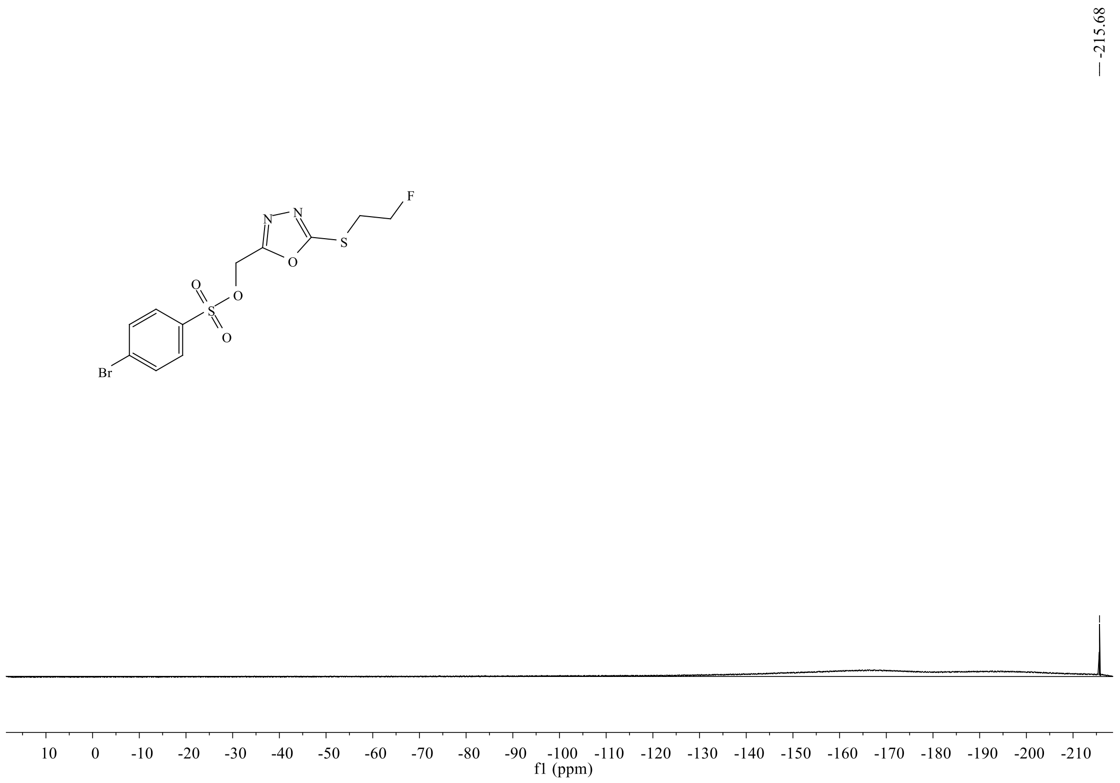

^{19}F NMR of compound **4a-16**

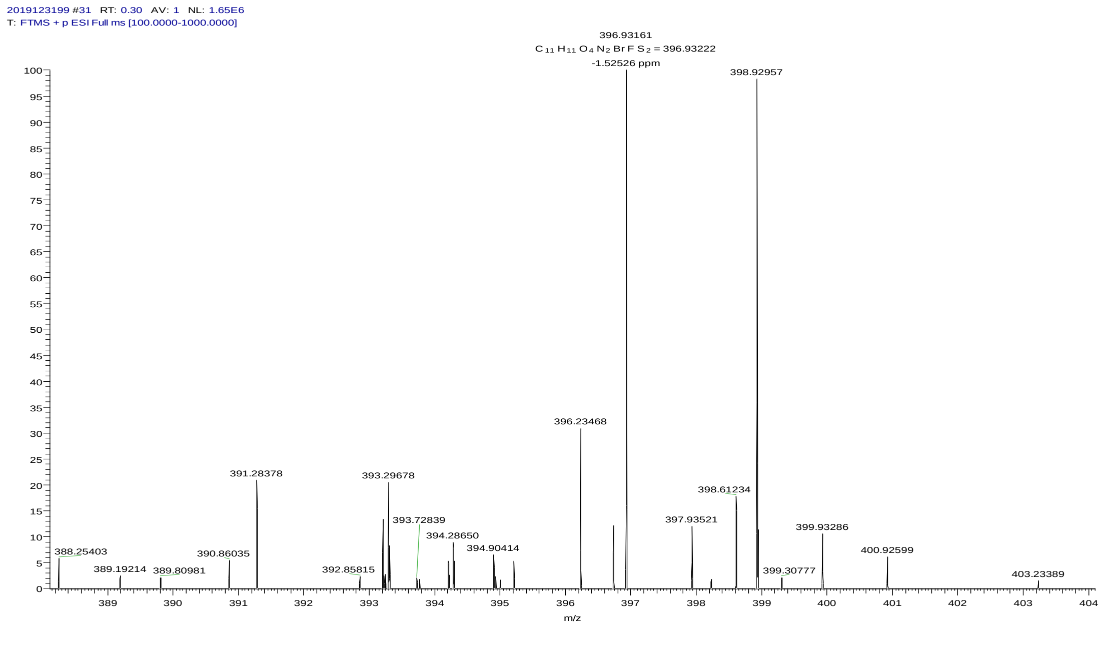

HRMS of compound **4a-16**

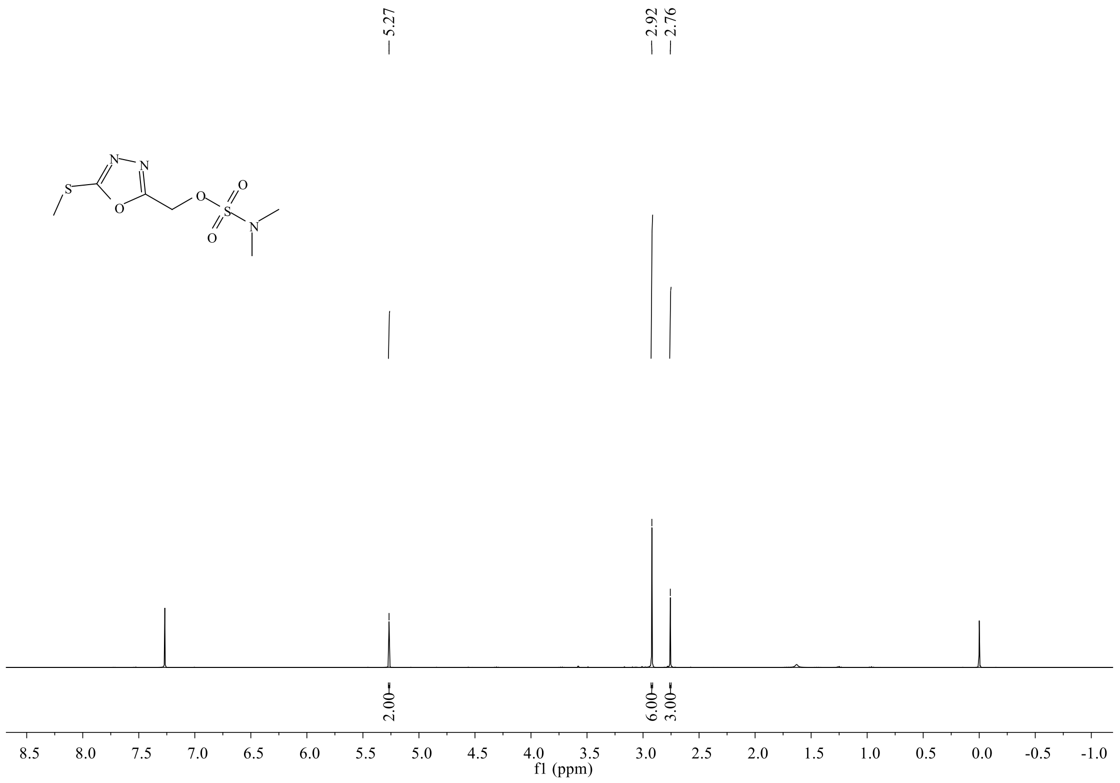

^{1}H NMR of compound **4a-17**

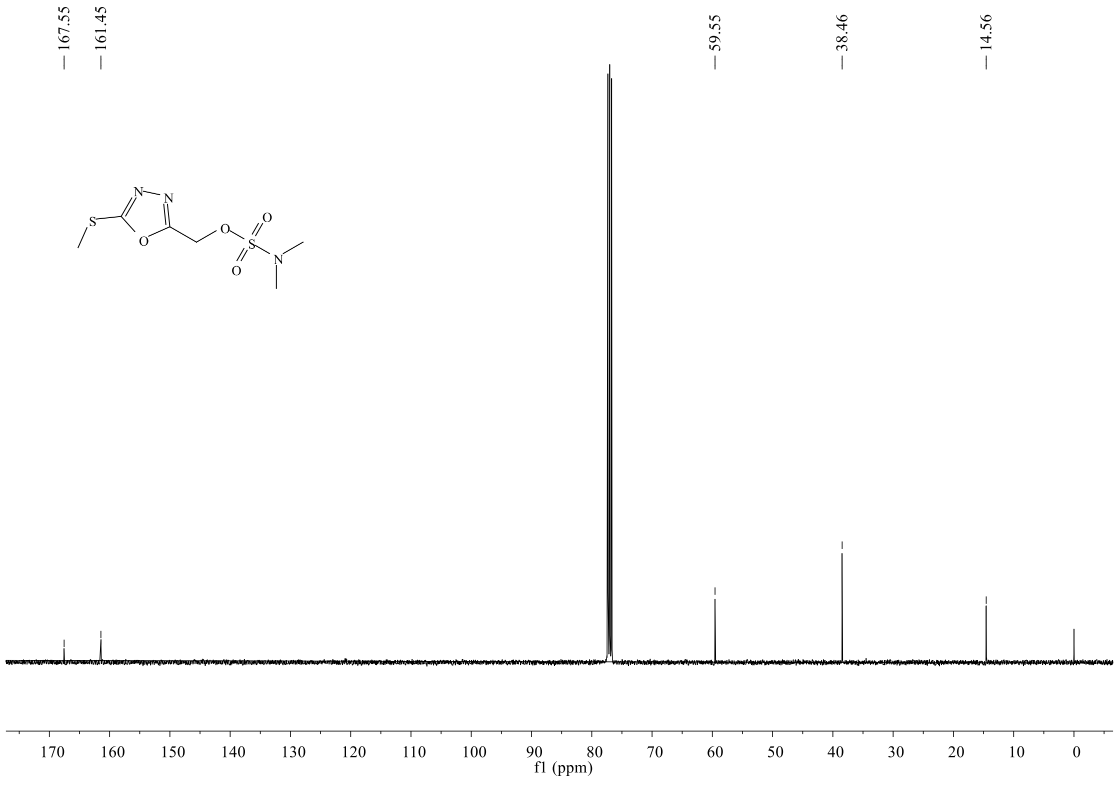

^{13}C NMR of compound **4a-17**

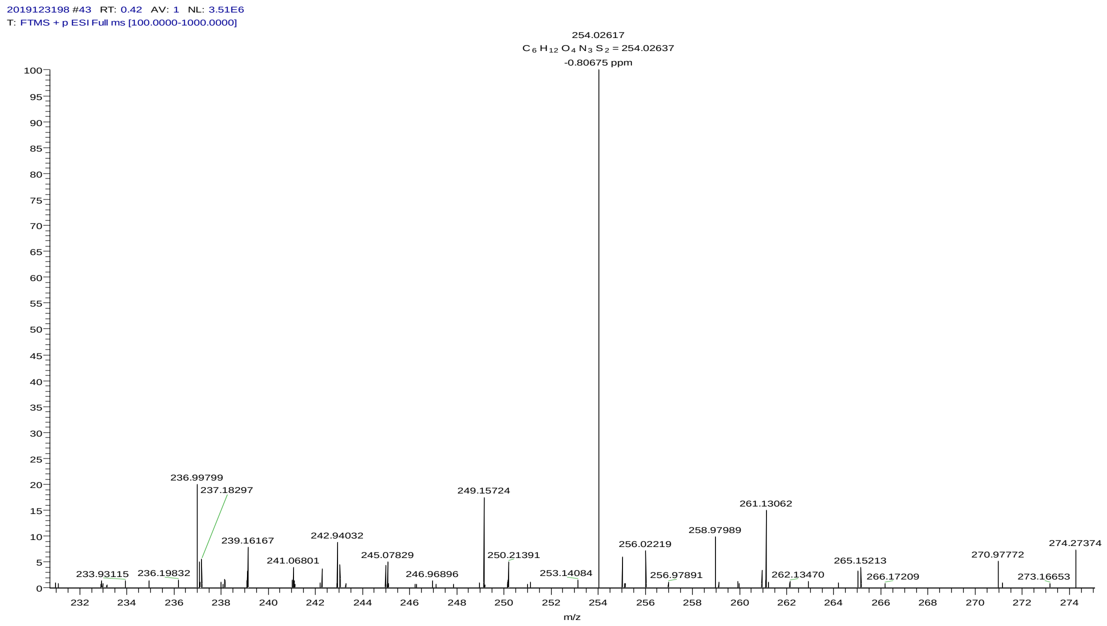

HRMS of compound **4a-17**

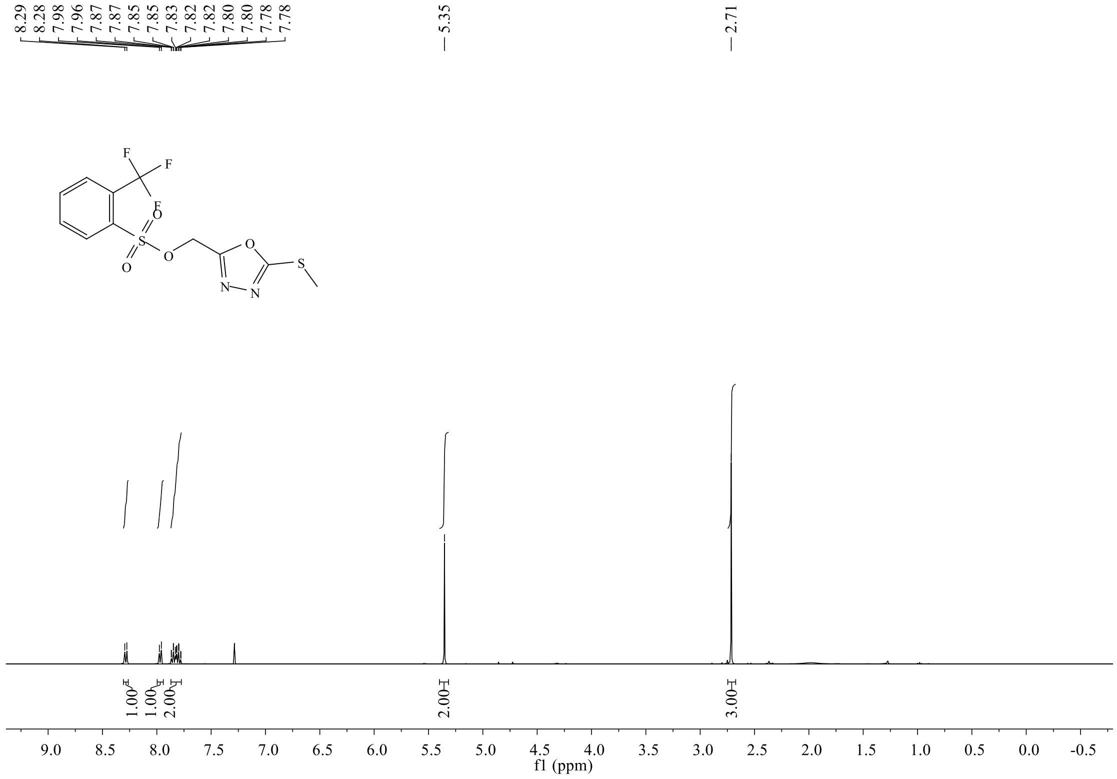

^{1}H NMR of compound **4a-18**

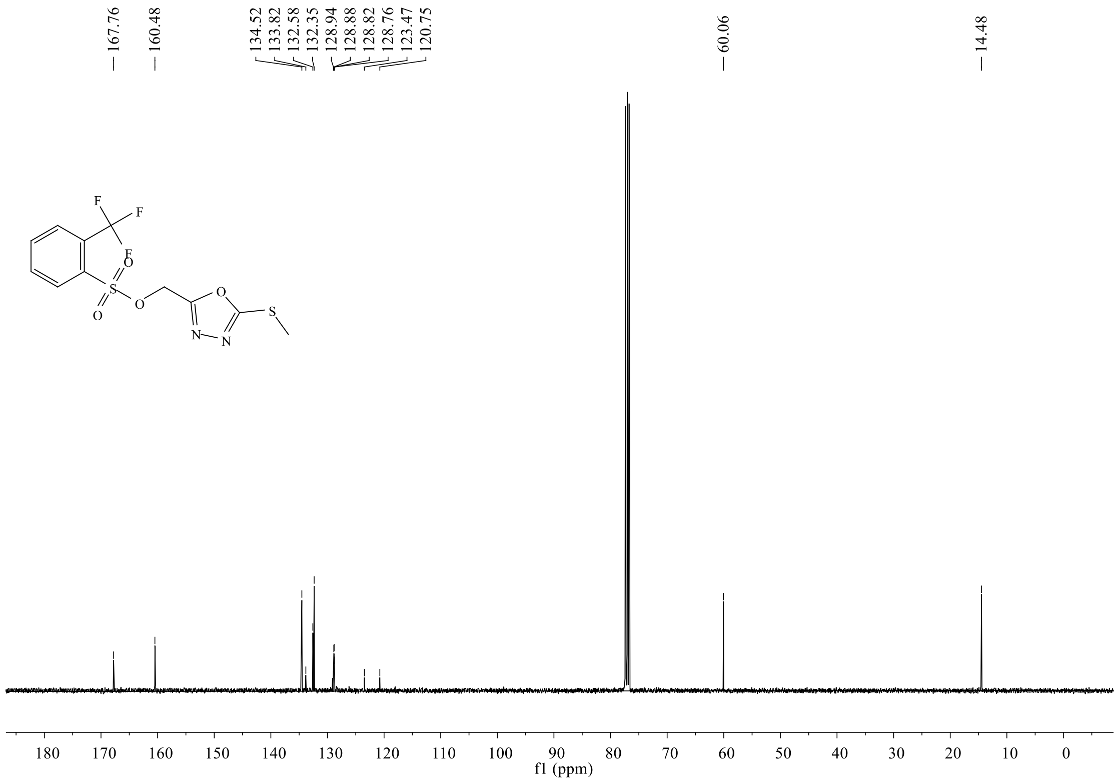

^{13}C NMR of compound **4a-18**

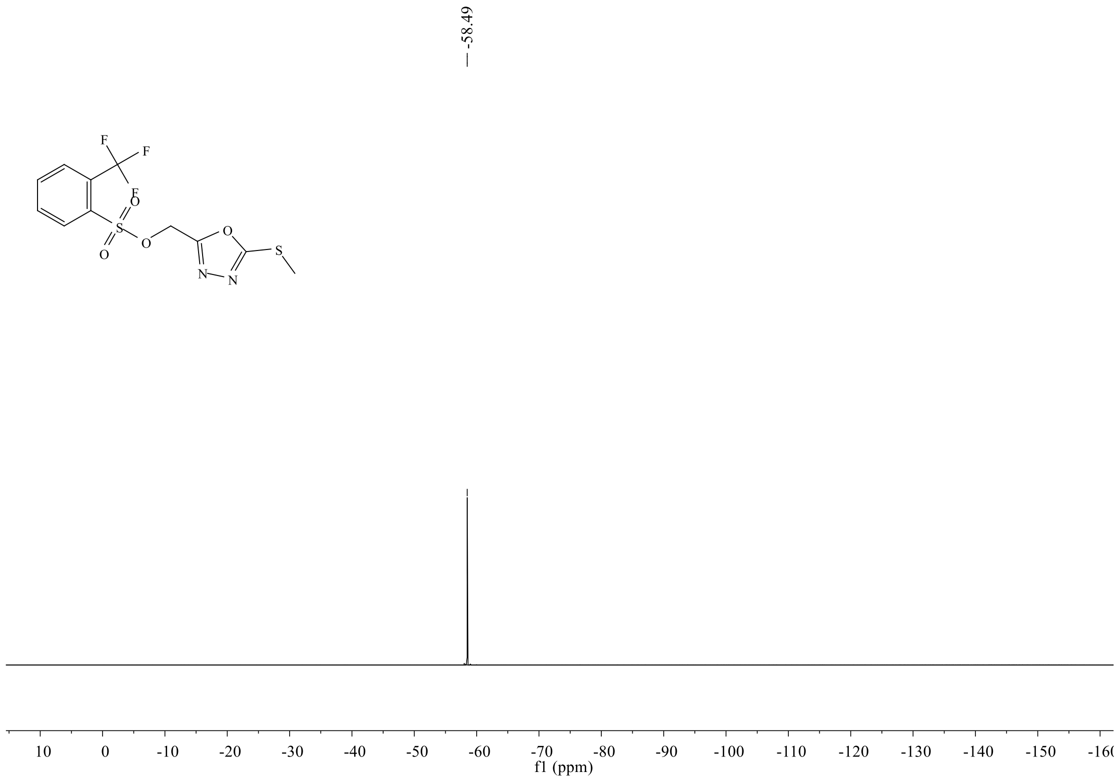

^{19}F NMR of compound **4a-18**

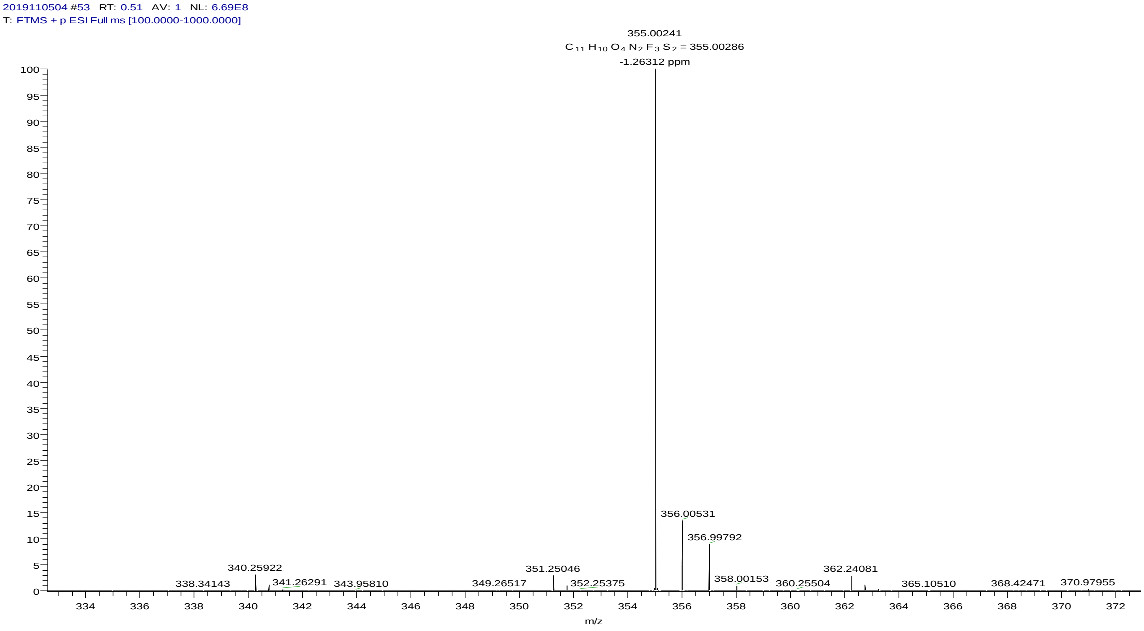

HRMS of compound **4a-18**

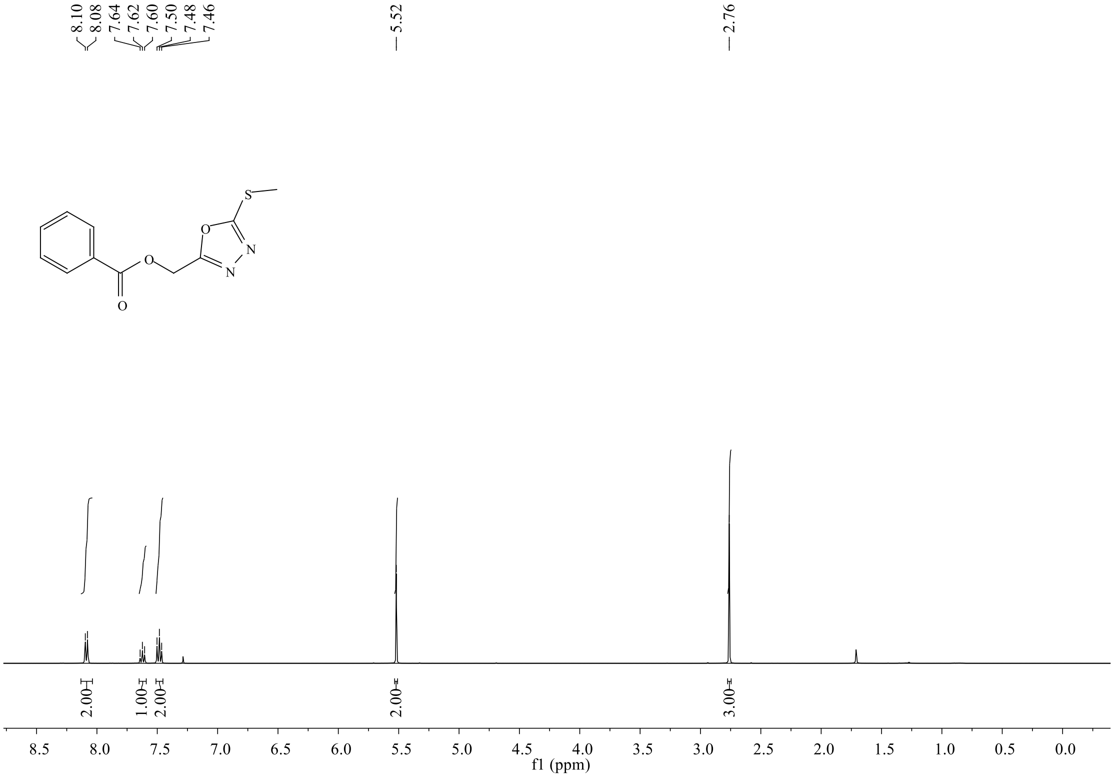

^{1}H NMR of compound **5a-1**

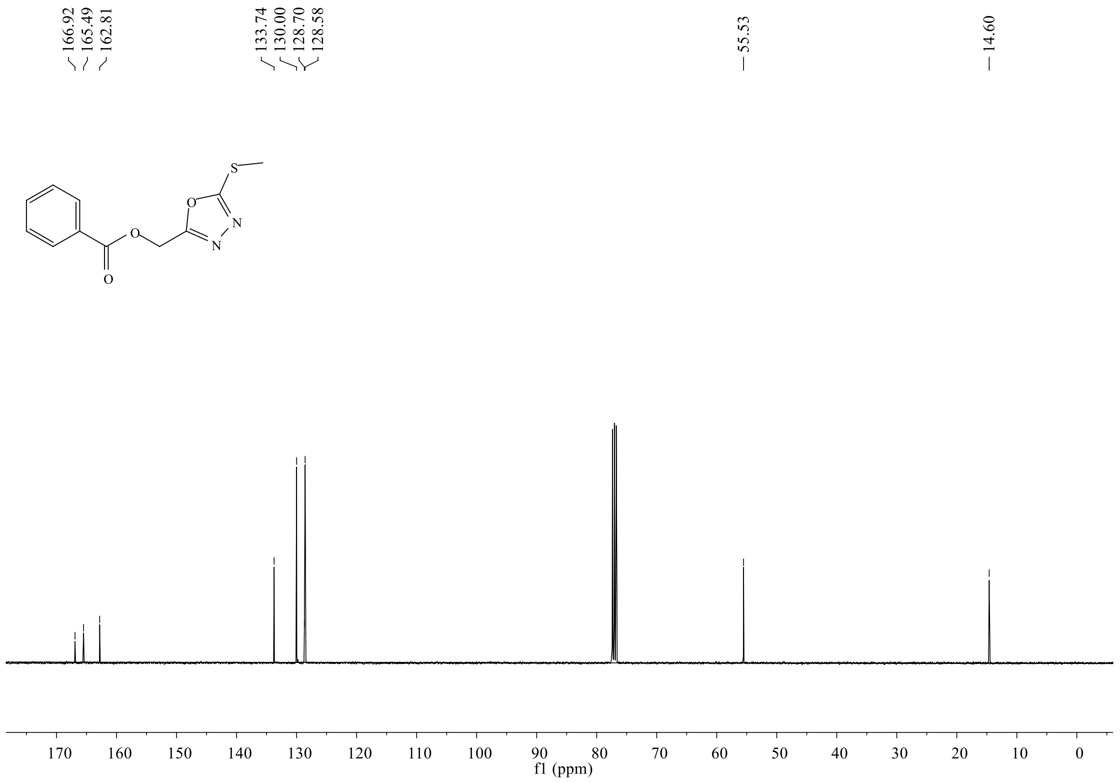

^{13}C NMR of compound **5a-1**

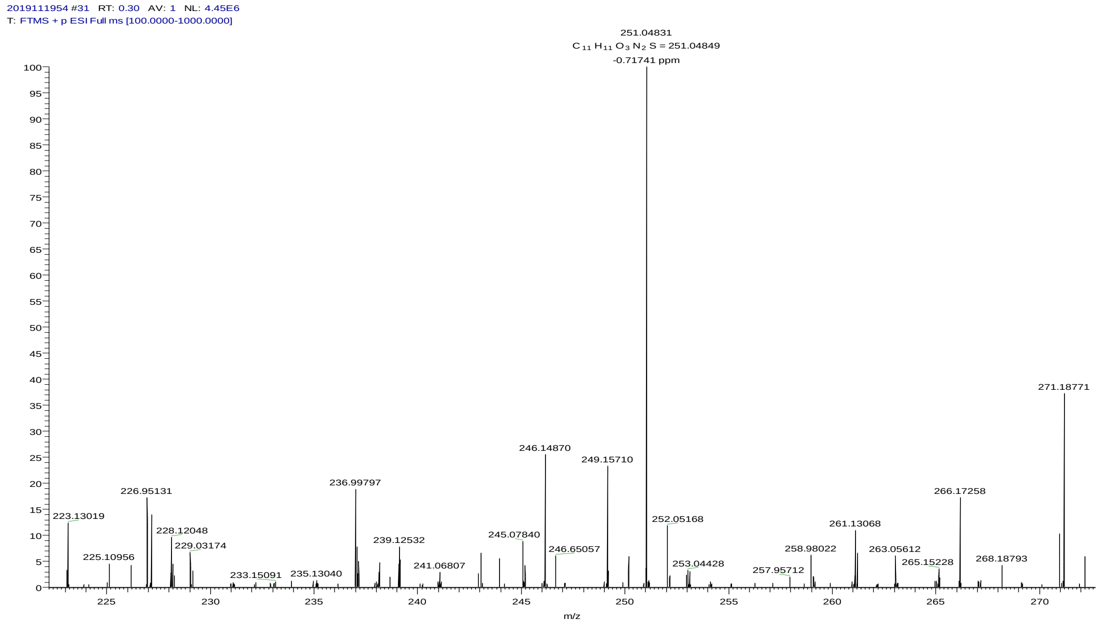

HRMS of compound **5a-1**

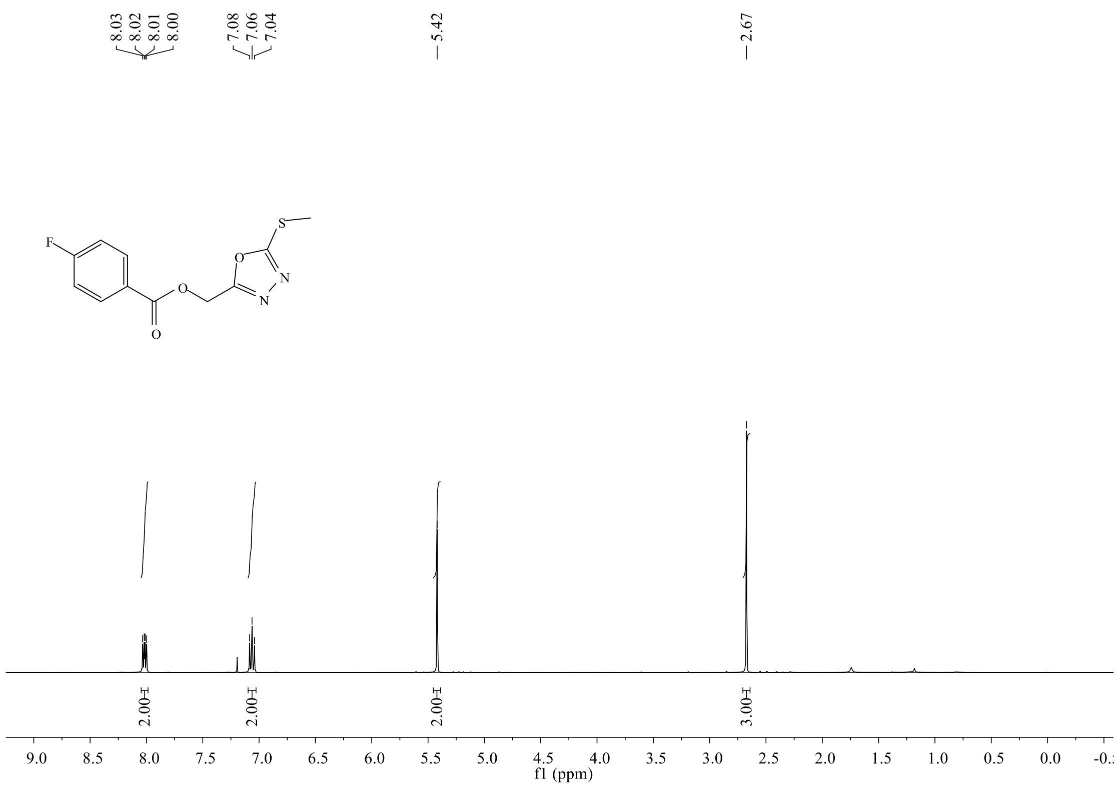

^{1}H NMR of compound **5a-2**

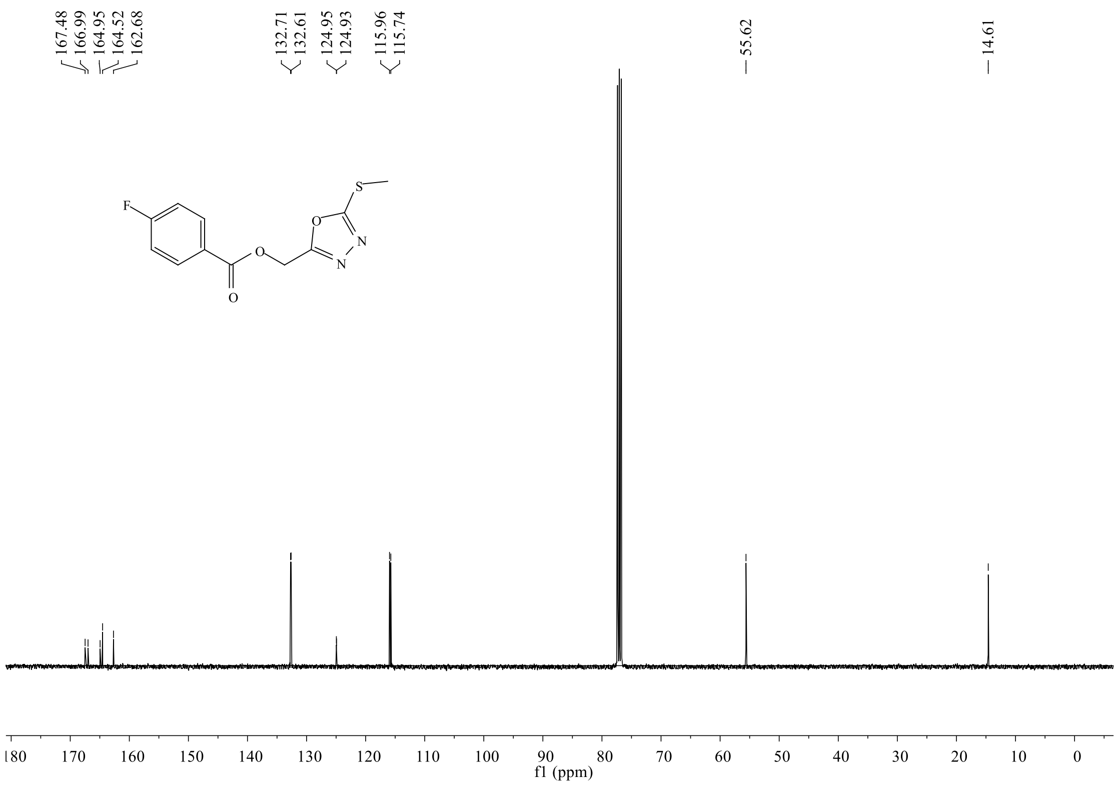

^{13}C NMR of compound **5a-2**

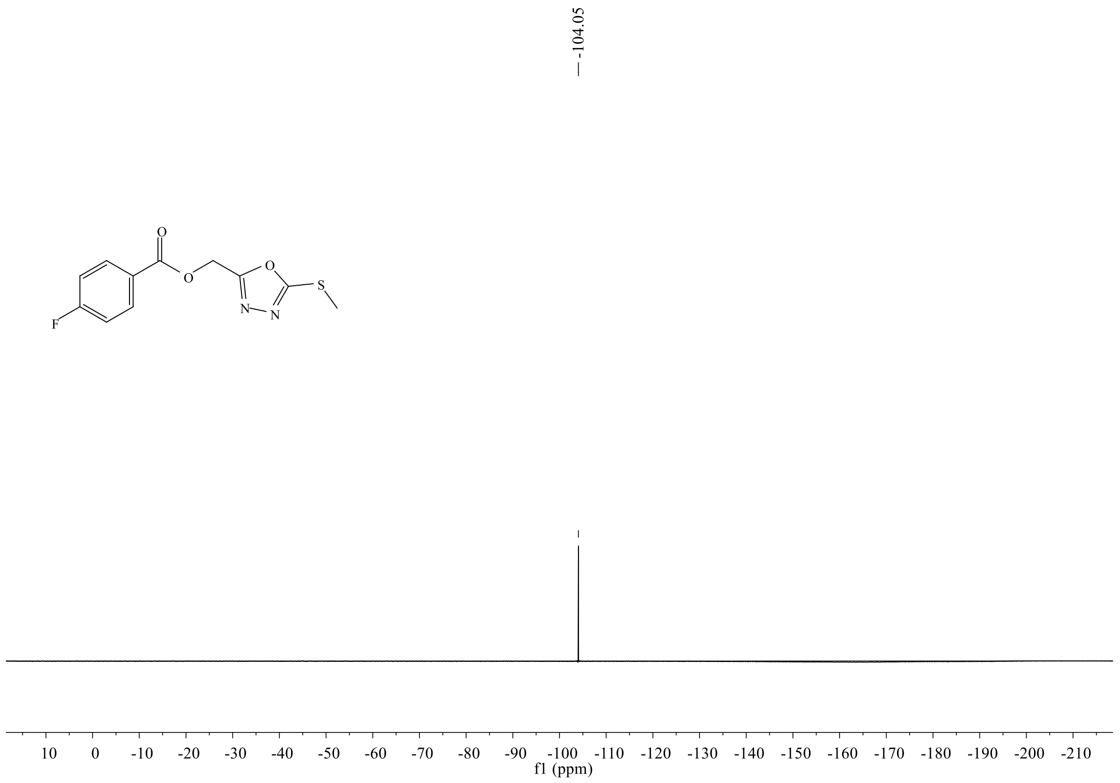

^{19}F NMR of compound **5a-2**

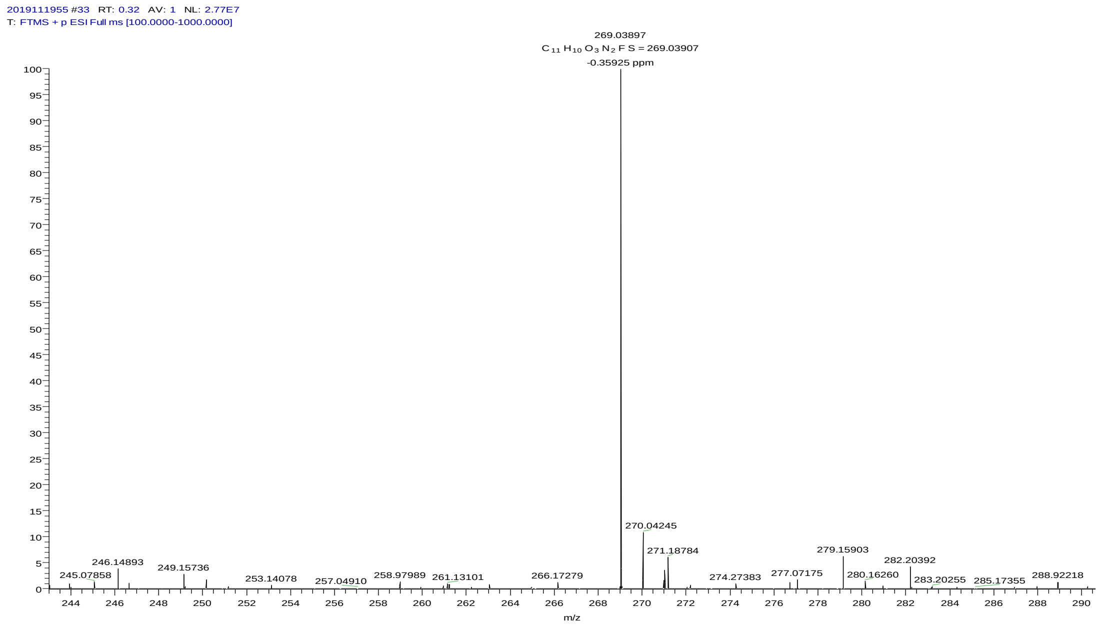

HRMS of compound **5a-2**

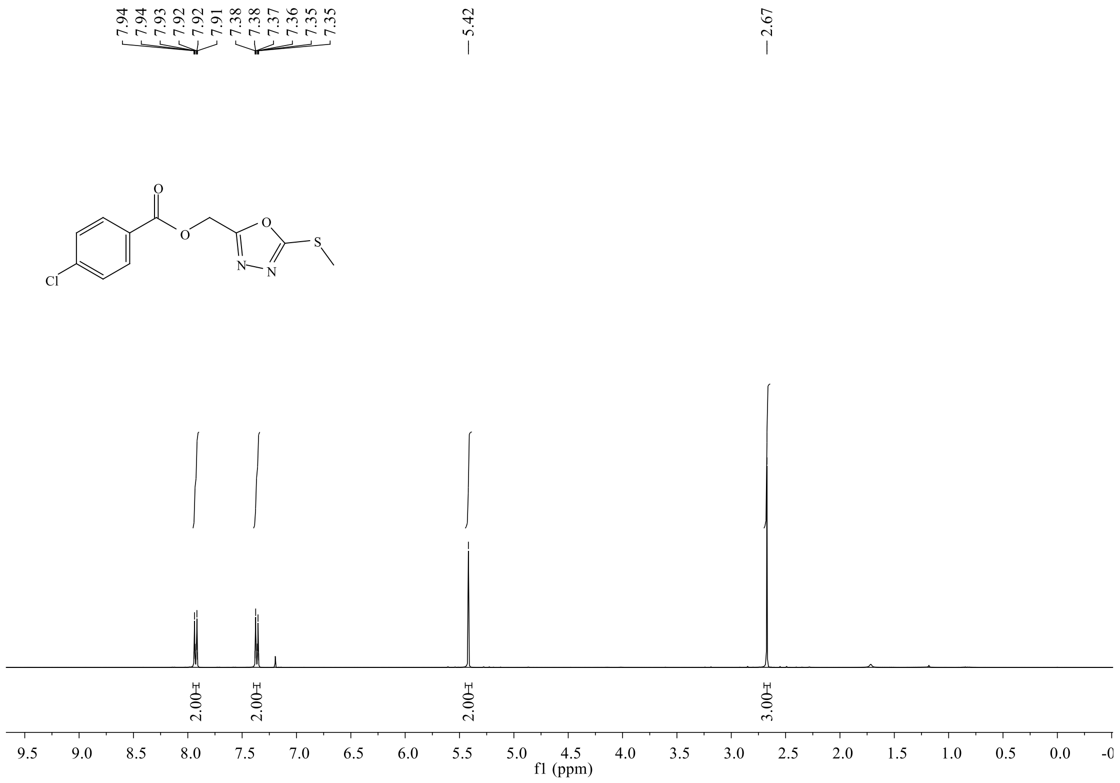

^{1}H NMR of compound **5a-3**

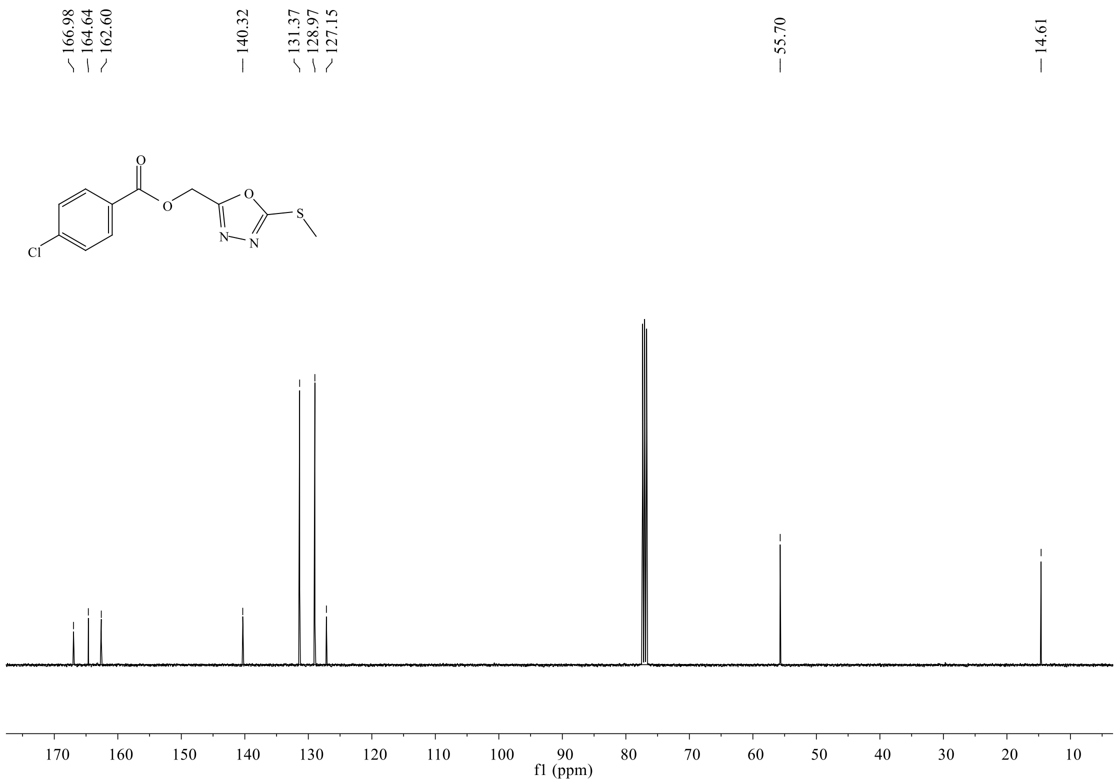

^{13}C NMR of compound **5a-3**

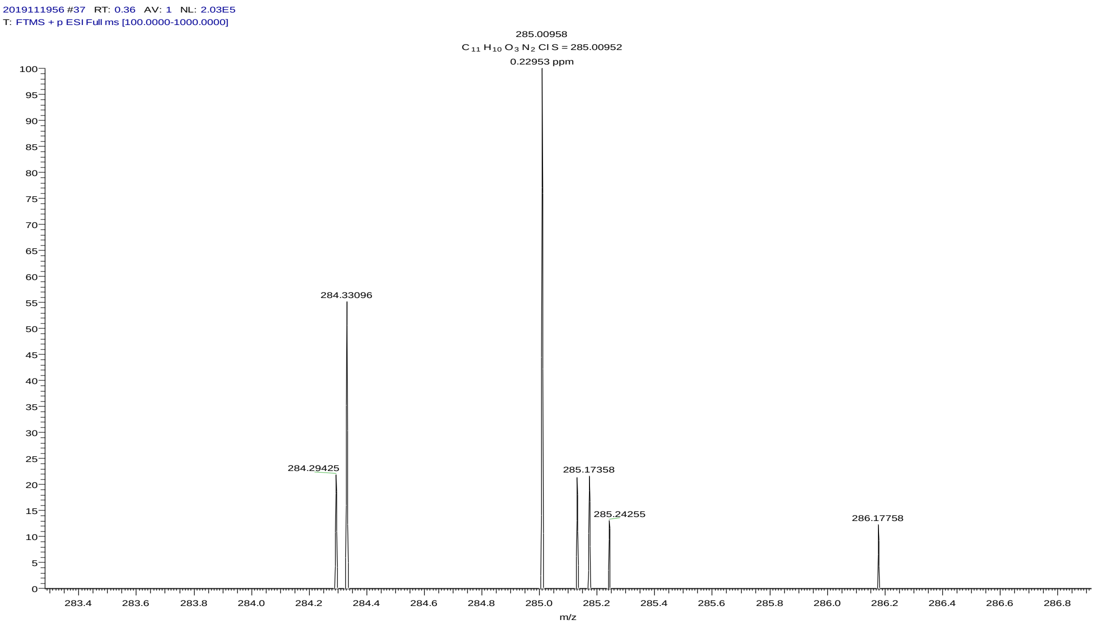

HRMS of compound **5a-3**

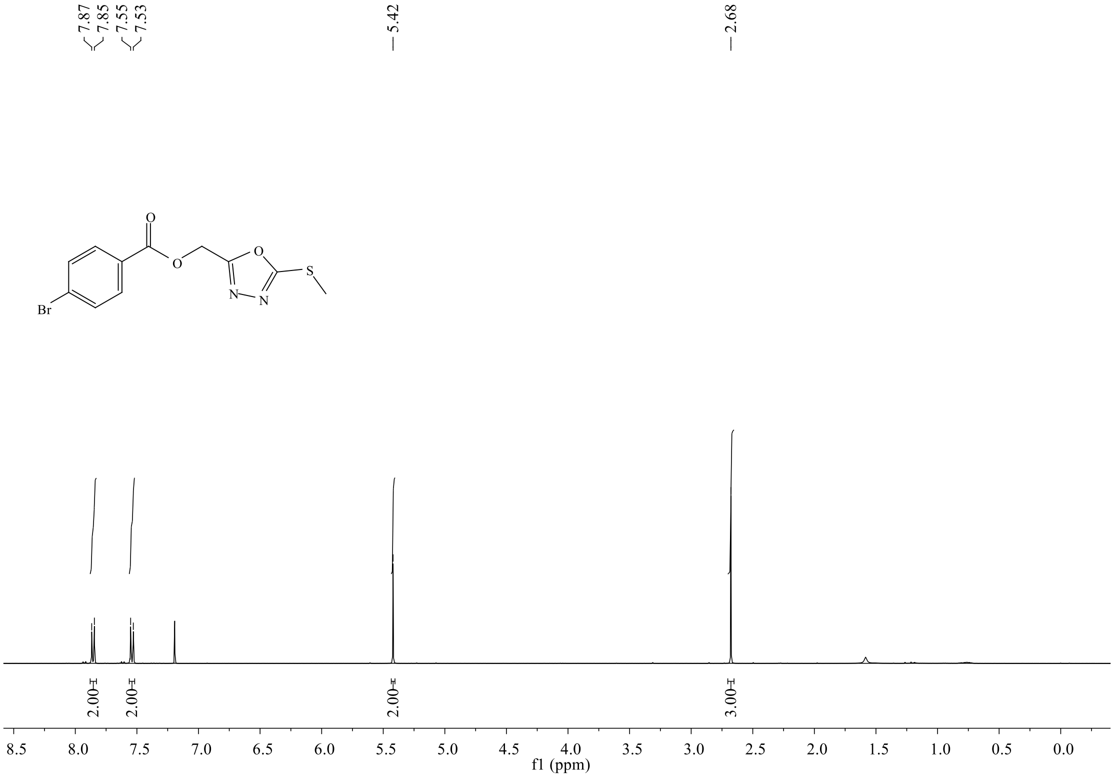

^{1}H NMR of compound **5a-4**

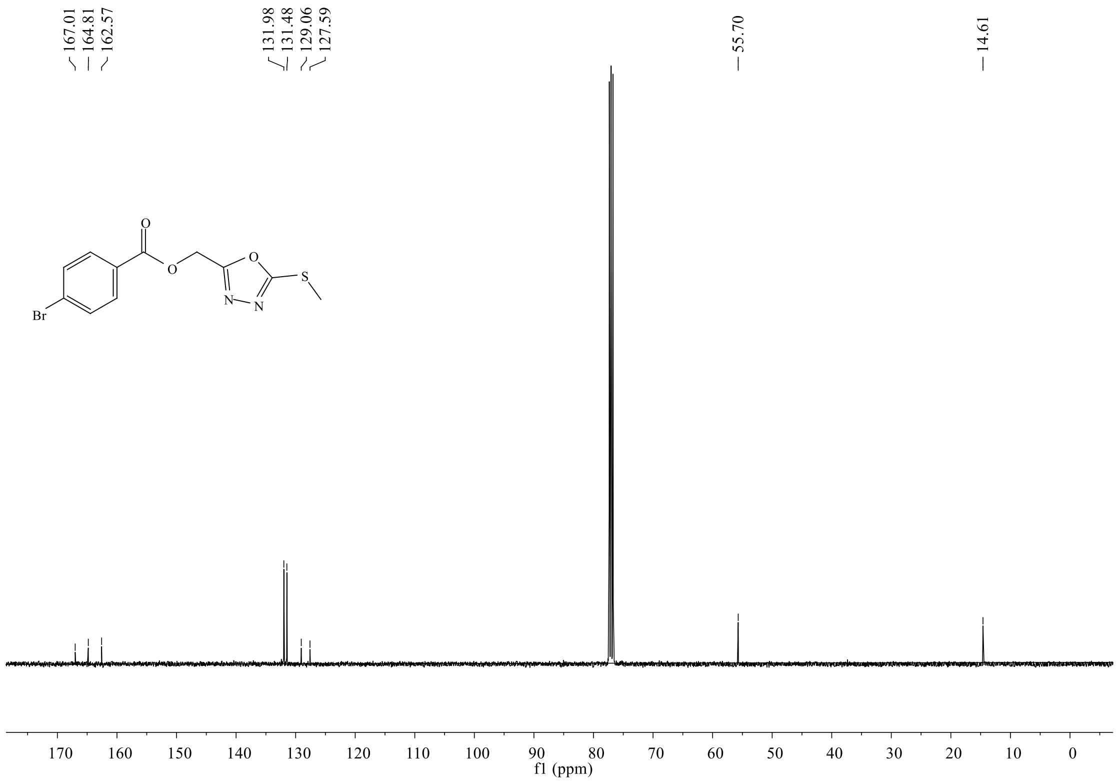

^{13}C NMR of compound **5a-4**

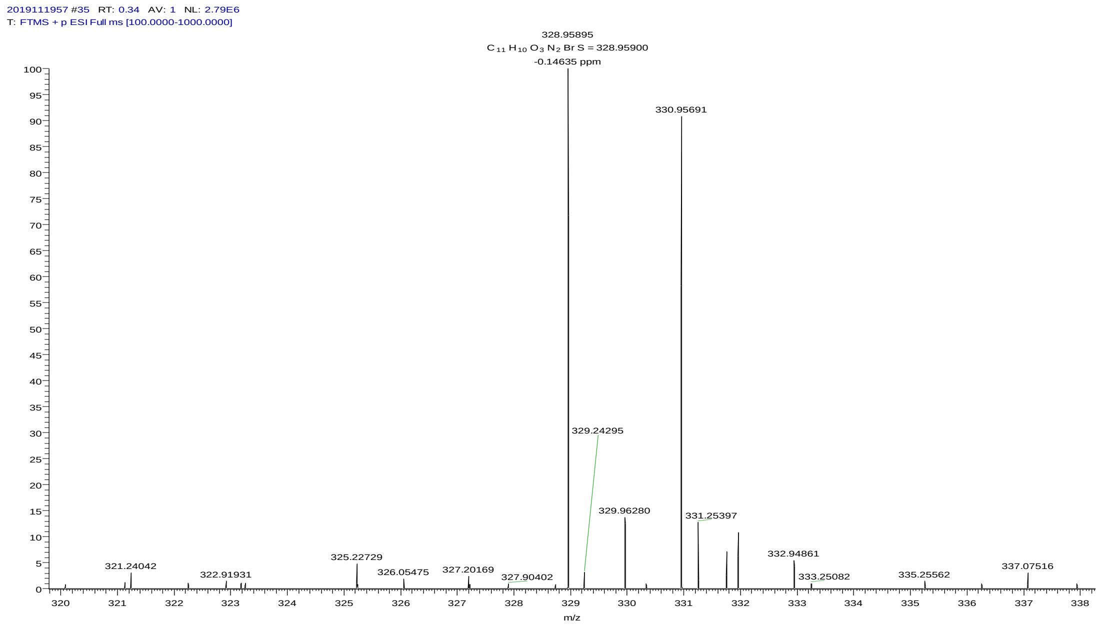

HRMS of compound **5a-4**

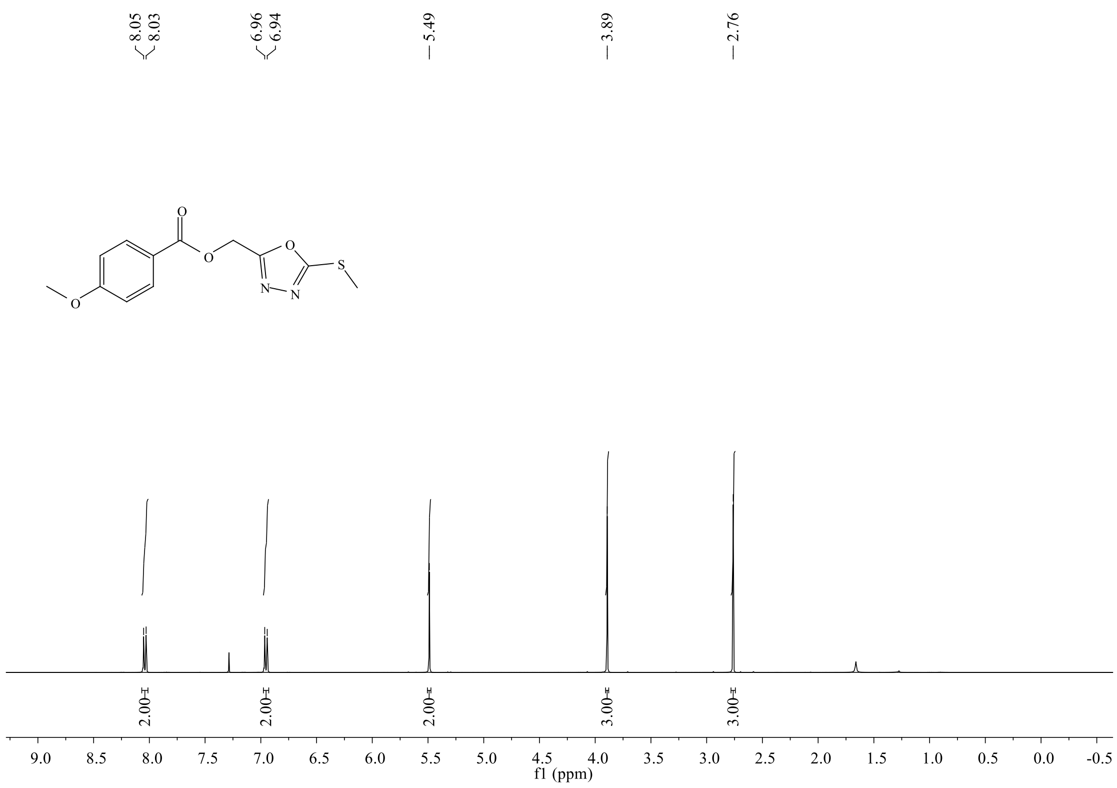

^{1}H NMR of compound **5a-5**

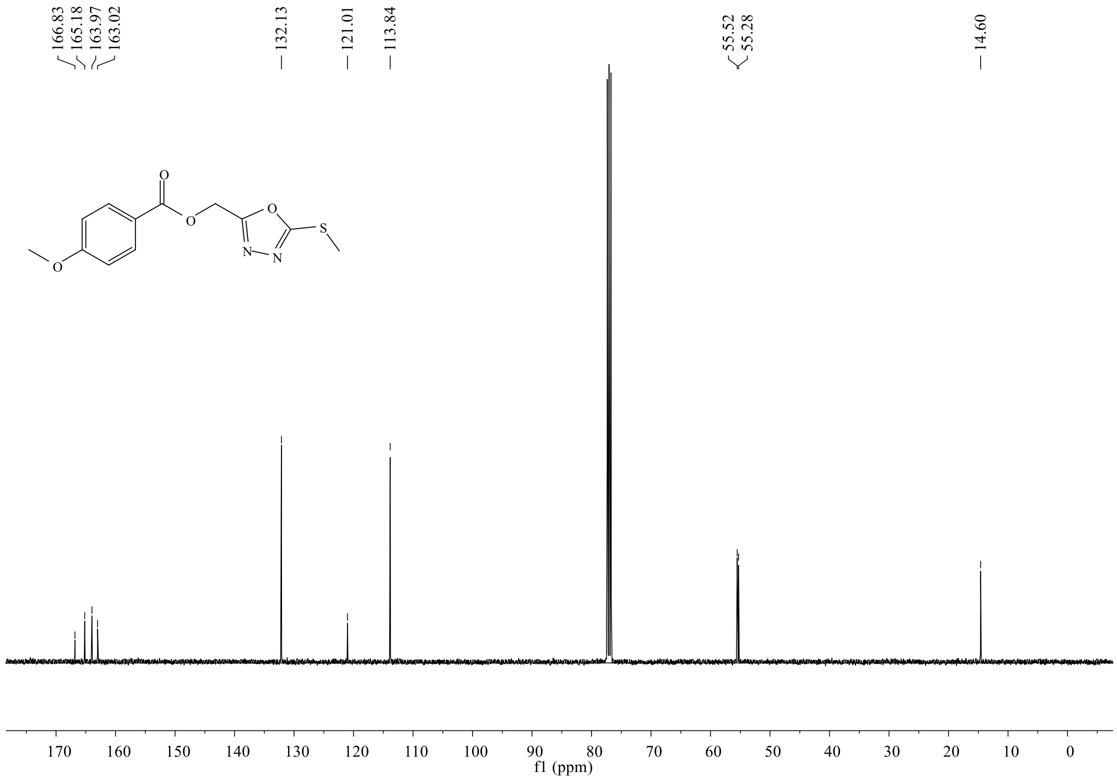

^{13}C NMR of compound **5a-5**

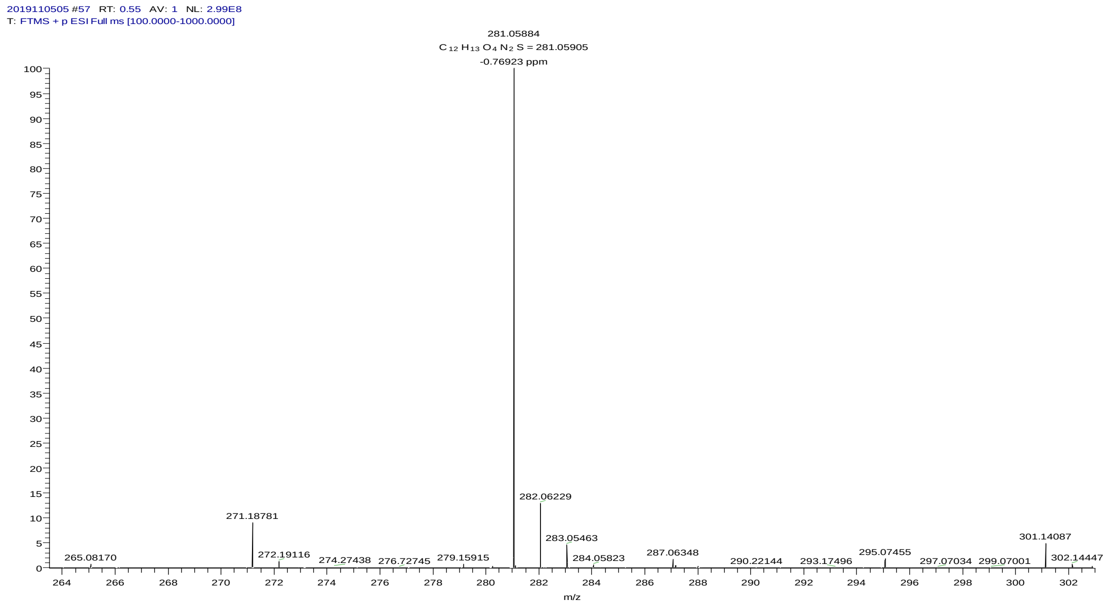

HRMS of compound **5a-5**

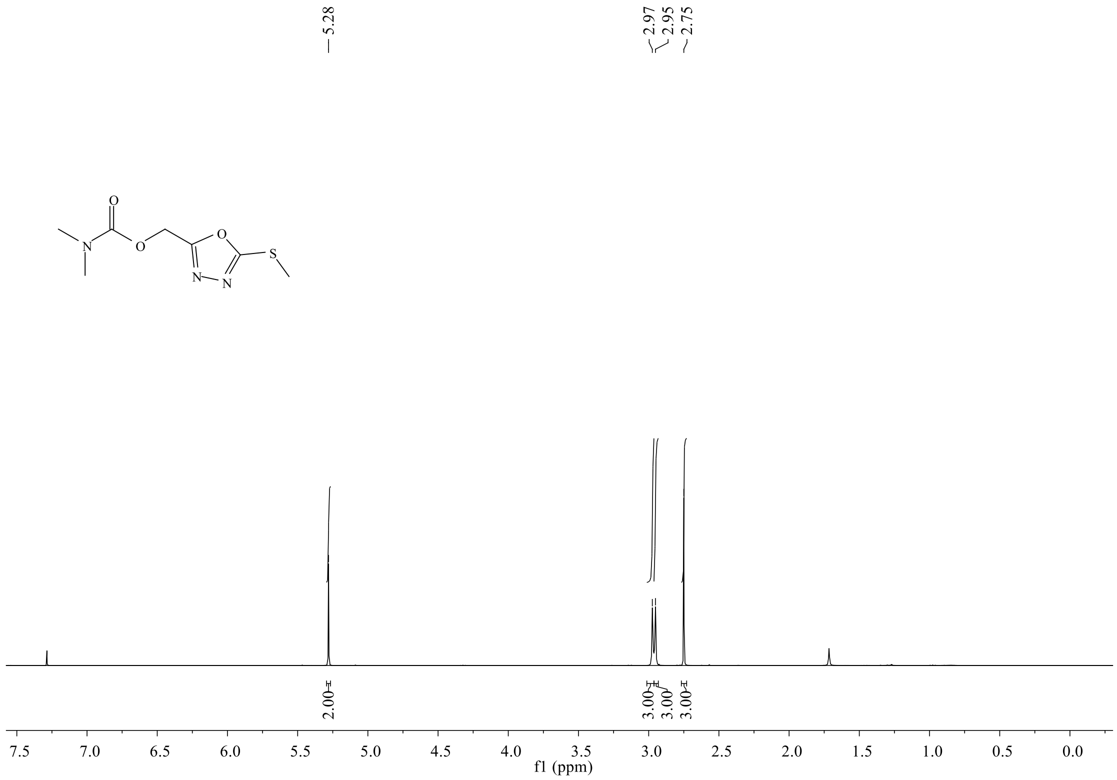

^{1}H NMR of compound **5a-6**

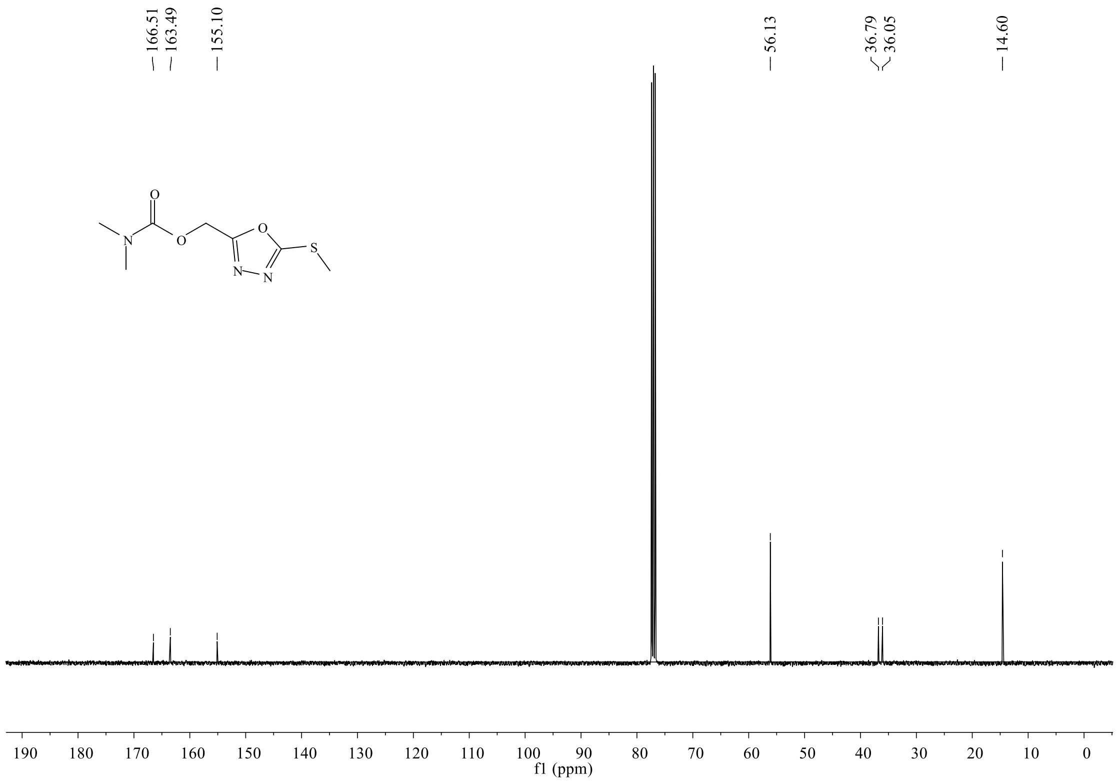

^{13}C NMR of compound **5a-6**

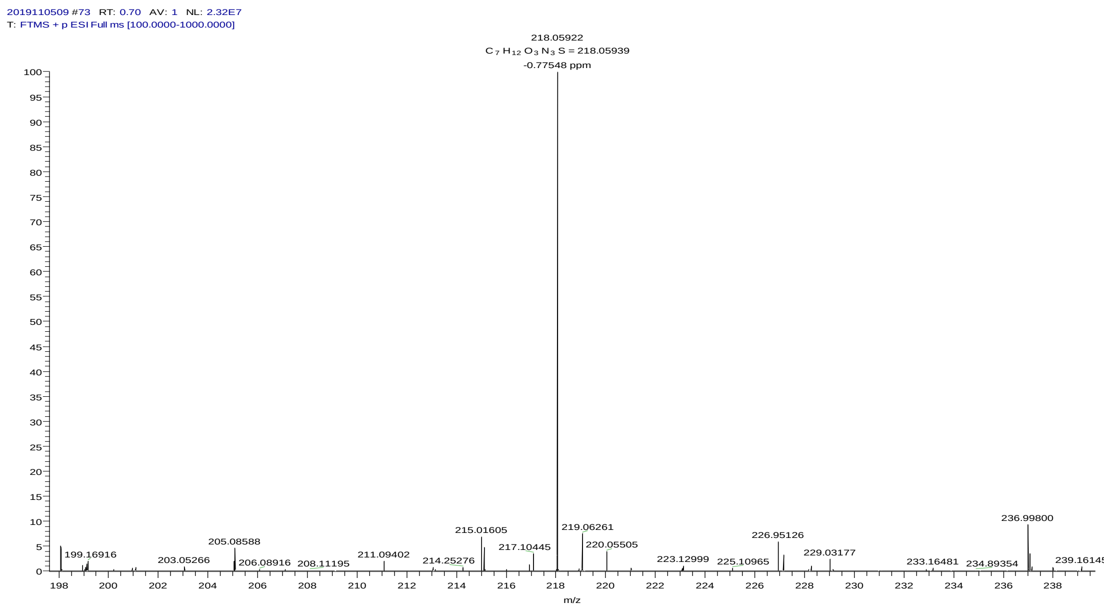

HRMS of compound **5a-6**
